# Using a Dual CRISPR/Cas9 Approach to Gain Insight into the Role of LRP1B in Glioblastoma

**DOI:** 10.3390/ijms241411285

**Published:** 2023-07-10

**Authors:** Joana Peixoto, Catarina Príncipe, Ana Pestana, Hugo Osório, Marta Teixeira Pinto, Hugo Prazeres, Paula Soares, Raquel T. Lima

**Affiliations:** 1 i3S—Instituto de Investigação e Inovação em Saúde, Universidade do Porto, 4200-135 Porto, Portugal; jpeixoto@ipatimup.pt (J.P.);; 2 Cancer Signaling and Metabolism Group, IPATIMUP—Institute of Molecular Pathology and Immunology of the University of Porto, Rua Alfredo Allen 208, 4169-007 Porto, Portugal; 3 Faculty of Sciences, University of Porto, 4169-007 Porto, Portugal; 4 IPATIMUP—Institute of Molecular Pathology and Immunology of the University of Porto, 4200-135 Porto, Portugal; 5 FMUP—Department of Pathology, Faculty of Medicine, University of Porto, Alameda Prof. Hernâni Monteiro, 4200-319 Porto, Portugal

**Keywords:** LRP1B, CRISPR/Cas9, glioblastoma, ploidy, secretome

## Abstract

*LRP1B* remains one of the most altered genes in cancer, although its relevance in cancer biology is still unclear. Recent advances in gene editing techniques, particularly CRISPR/Cas9 systems, offer new opportunities to evaluate the function of large genes, such as *LRP1B*. Using a dual sgRNA CRISPR/Cas9 gene editing approach, this study aimed to assess the impact of disrupting *LRP1B* in glioblastoma cell biology. Four sgRNAs were designed for the dual targeting of two *LRP1B* exons (1 and 85). The U87 glioblastoma (GB) cell line was transfected with CRISPR/Cas9 PX459 vectors. To assess *LRP1B*-gene-induced alterations and expression, PCR, Sanger DNA sequencing, and qRT-PCR were carried out. Three clones (clones B9, E6, and H7) were further evaluated. All clones presented altered cellular morphology, increased cellular and nuclear size, and changes in ploidy. Two clones (E6 and H7) showed a significant decrease in cell growth, both in vitro and in the in vivo CAM assay. Proteomic analysis of the clones’ secretome identified differentially expressed proteins that had not been previously associated with *LRP1B* alterations. This study demonstrates that the dual sgRNA CRISPR/Cas9 strategy can effectively edit *LRP1B* in GB cells, providing new insights into the impact of *LRP1B* deletions in GBM biology.

## 1. Introduction

Glioblastoma (GB) is a highly malignant brain tumor known for its aggressive characteristics, including angio-invasive behavior and resistance to conventional treatments [1,2]. Despite the current standard of care, the Stupp protocol, which combines maximal safe surgical tumor resection with radiotherapy and chemotherapy using temozolomide, the prognosis for GB patients remains extremely poor. The median overall survival (OS) for patients receiving standard therapy is 15−18 months, dropping to only 3−4 months for untreated patients [3,4]. This highlights the critical need to explore novel approaches to understand the biology of GB, which may provide clinically relevant strategies to improve patient outcomes [4].

Over the years, and even with the increased knowledge brought by next-generation gene sequencing, the *LRP1B* gene remains one of the most frequently altered genes among different cancers [5,6,7,8,9,10]. In GB in particular, *LRP1B* deletions are associated with poorer patient outcomes [11], although its functional relevance remains unknown, as for other tumor settings. The *LRP1B* gene is located on chromosome 2 and encodes for a giant LRP1B protein, a cell surface receptor, member of the LDL receptor family, which plays a crucial role in various cellular processes, including endocytosis, cellular signaling, and clearance of extracellular molecules [6,7,12]. LRP1B is broadly expressed in human tissues [6]; in particular, it is highly expressed in the brain, possibly indicating a pivotal role in brain physiology and function [13].

The study of LRP1B cellular function has been hindered by several major obstacles. At 1.9 MB, 4599 amino acids, and 515 kDa, LRP1B is one of the largest encoded proteins in the human genome, making both its full expression and its effective silencing challenging in cell lines. [6,14]. Still, important knowledge on LRP1B has been gathered mostly from studies carried out in cancer cell lines, either by partially re-establishing its expression (with mini-receptors) [12,15,16,17] or by downregulating its expression through RNA interference [17,18,19,20]. These studies enabled the description of LRP1B as a putative tumor suppressor in several tumors, the identification of some of its ligands, the investigation of interactions with signaling pathways, and the exploration of some of its potential roles in tumorigenesis (as reviewed in [6]). Moreover, it has been shown that LRP1B’s expression influences the uptake of liposomal drugs, affecting the response to drugs, such as pegylated liposomal doxorubicin [16,17]. In addition, the endocytic activity of LRP1B plays a role in the crosstalk between tumor cells and the microenvironment, as a secretome modulator, and influences cellular processes such as angiogenesis and metastasis [12,18,20].

The evaluation of *LRP1B* alterations at a genetic level is yielding interesting results in terms of their impact on cancer patient prognosis and response to therapy. Besides *LRP1B* mutations associating with prognosis/survival in cancer patients, including GB [9,11,21,22], *LRP1B* mutations have been associated with higher tumor mutation burden (TMB) and better prognosis in lung cancer and melanoma patients [10]. *LRP1B* expression is positively associated with immune cell marker genes [9,10,21,22,23,24]. Accordingly, *LRP1B* mutations are associated with favorable outcomes in response to immune checkpoint inhibitors (ICI) across multiple cancer types, similar to the higher TMB cancer outcomes [10,25]. Nevertheless, other studies have shown that the *LRP1B* mutations are associated with a worse prognosis in hepatocellular carcinoma (HCC) patients and with a poor response to ICI, being also related to TMB and immune infiltration [21,26].

Despite all the studies pointing towards LRP1B as a prognosis and/or predictive response biomarker and as a possible target in cancer treatment, the full impact of LRP1B alterations at gene and protein level on cancer biology remains unknown. To fully comprehend the significance of LRP1B in cancer, it is crucial to investigate its functional implications using suitable biological models that may allow the evaluation of the complex nature of LRP1B alterations and their specific effect.

Recent advances in gene editing techniques utilizing CRISPR/Cas9 have provided researchers with new tools for investigating gene and protein function [27,28,29]. The ability to edit the *LRP1B* gene and effectively impact protein expression may be crucial to unravel the role of LRP1B in cancer biology and to identify new players. To the best of our knowledge, only one study presenting a lentiviral CRISPR/Cas9 system for LRP1B knockdown has been published, mainly focusing on its effect on lipid metabolism in HCC [30].

Therefore, our study aimed to develop *LRP1B*-silenced GB cell lines using CRISPR/Cas9 technology and further investigate the impact of *LRP1B* gene editing on GB cell biology and growth in vitro and in vivo. Additionally, we aimed to gain a deeper understanding of the potential crosstalk between LRP1B and the tumor microenvironment by evaluating the secretome of GB LRP1B-altered cells. This study provides a better understanding of the role of LRP1B in GB and opens new opportunities for future research.

## 2. Results

### 2.1. Dual sgRNA CRISPR/Cas9-Mediated Strategy to Target LRP1B

**Figure 1:**
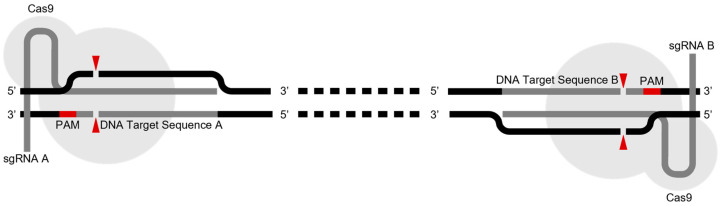
Schematic representation of the dual sgRNA CRISPR/Cas9 deletion strategy for one exon. Cas9 nuclease (gray) complexed with a sgRNA (dark gray) recognizes and binds to the protospacer adjacent motif (PAM) sequence (red). PAM binding enables local DNA strand separation and subsequent base pairing between the sgRNA target sequence and its complementary DNA strand. Successful base pairing induces Cas9 activation and leads to a double-strand break (DSB). The red arrowheads indicate the predicted Cas9 cleavage sites (3 bp upstream (5′) of the PAM sequence) within the target DNA. Simultaneous cleavage of both target sites, followed by nonhomologous end-joining (NHEJ) repair, often results in the deletion of the intervening DNA through the re-ligation of the Cas9-induced distal blunt ends.

Our first aim was to generate LRP1B knockout cells using the CRISPR/Cas 9 strategy. Given the enormous size of the *LRP1B* gene [6,7], we employed a dual sgRNA CRISPR/Cas9 deletion strategy to increase the potential of more efficient abrogation (Figure 1). This strategy uses two sgRNAs to direct the activity of Cas9 to the sites flanking one target region, causing two double-strand breaks (DSBs) in the same region, which are then frequently repaired through the nonhomologous end-joining (NHEJ) pathway.

To select which *LRP1B* gene region to target, we comparatively analyzed all putative alternatively spliced protein-coding transcripts of the gene (as shown in Supplementary Figure S1) [31,32,33]. According to EMBL-EBI’s Ensembl/GENCODE database, the *LRP1B* gene has the potential to generate four predicted protein-coding transcripts based on bioinformatics analysis, although only one has been biologically described so far (referred to as *LRP1B transcript 1*; Supplementary Figure S1 [33,34,35]. Usually, a more efficient permanent gene loss/function is achieved when the targeted region is at the most upstream exon (which is common to all predicted transcripts of the target gene) [36]. However, since there was no unique exon conserved among all the predicted transcripts of *LRP1B*, we decided to target both exons 1 and 85, which were shared by most of the predicted transcripts.

**Figure 2:**
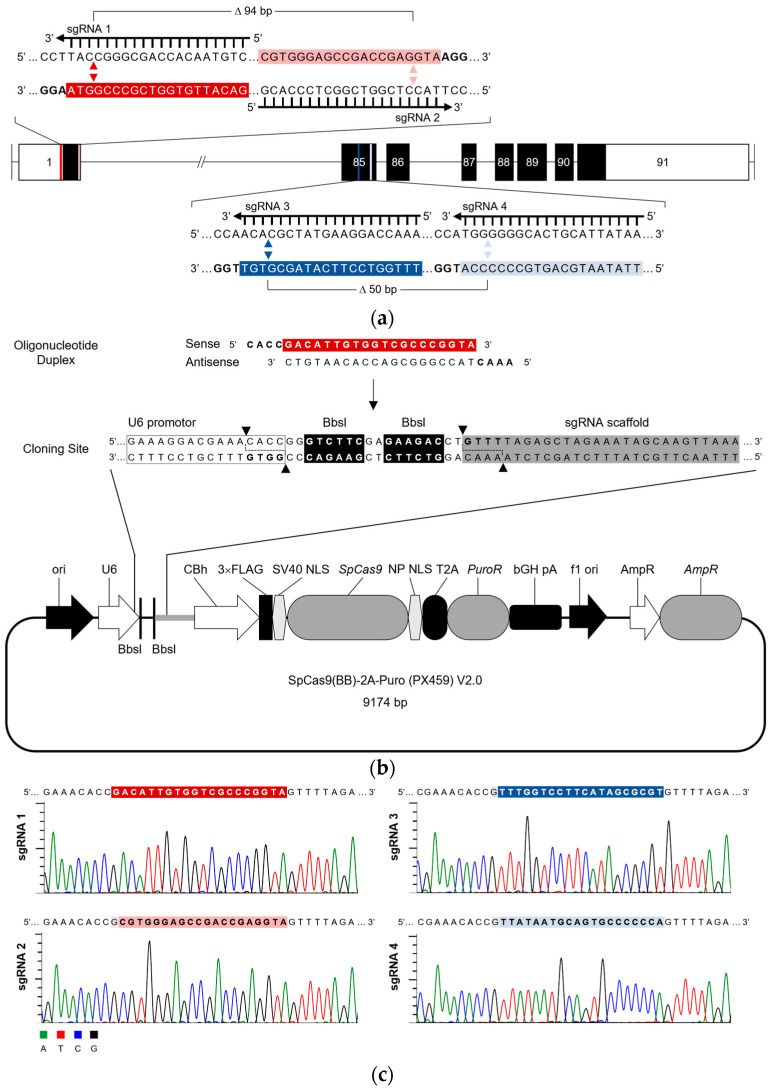
Creation of the sgRNA/Cas9 expression vectors to target human *LRP1B* gene at exons 1 and 85. (**a**) Schematic representation of our dual sgRNAs targeting the human *LRP1B* gene at exons 1 and 85. Boxes on the horizontal connecting black line (introns) represent exons. Filled black boxes (or portion of boxes) represent coding sequence, and the unfilled (white) portions of boxes represent non-coding sequence (i.e., untranslated region). CRISPR/Cas9 target sites are shown as colorful bars. sgRNA target sequences (highlighted in color) and their respective PAM sequences (bold) are also represented. Colorful arrowheads mark the predicted Cas9 cleavage sites, (3 bp upstream (5’) of the PAM sequences) within the target exons. Predicted CRISPR/Cas9-mediated deletions (Δ, bp) are also indicated. The specific oligonucleotide sequences for each sgRNA cloning are presented in Supplementary Table S3. (**b**) The sgRNA/Cas9 expression vectors generated. Schematic representation of the cloning of sgRNA-encoding oligonucleotide duplexes into the PX459 V2.0 vector. sgRNA-encoding oligonucleotide duplexes contain BbsI compatible with vector overhangs (bold). Sense-oriented oligonucleotide encodes the sgRNA target sequence (highlighted in red). BbsI digestion of the PX459 vector allows the direct insertion of each oligonucleotide duplex:recognition site (highlighted in color) and cleavage site (black arrowheads) of BbsI restriction enzyme are also represented. PX459 partial DNA sequence was retrieved from https://www.addgene.org/62988/sequences/ accessed on 3 April 2020), vector elements are shown in the correct order (not at the precise scale). (**c**) Representative Sanger sequencing electropherograms of the four sgRNA/Cas9 expression vectors (sgRNAs 1-4), presenting the correctly inserted sgRNA target sequences (sequence highlighted in color).

Once the targets were defined (exons 1 and 85), a set of sgRNA pairs was designed for each of them using the “Benchling CRISPR Guide RNA design” software. This tool screened for 20 nt sequences immediately preceding a canonical *Streptococcus pyogenes* Cas9 (SpCas9) PAM sequence 5′-NGG-3′ [27,37] and ranked them based on their predicted off-target score [37]. The selection of sgRNA pairs used was based on their on-target specificity but also considering the need to discard potential Cas9 off-target cleavage activity (and potentially related *off-target* effects) that would allow disrupting deletions to be easily screened through conventional PCR. Based on these criteria, the selected sgRNAs used in this study are presented in Figure 2a (and Supplementary Table S1). Additionally, an in silico assessment was conducted to evaluate potential off-target sequences within the human genome for each of the selected sgRNAs, which is provided in Supplementary Table S2.

In addition, and to potentiate cloning efficiency into the vector (PX459 V2.0) chosen for the delivery and expression of our sgRNA/CRISPR/Cas9 machinery in human tumor cells [38], specific alterations were introduced to the former sgRNAs (as shown in Figure 2b). Specifically, since PX459 uses the human U6 promoter (which favors the presence of a guanine at the transcription start site (+1) [39]), an extra guanine was included in the 5′ ends of sgRNA-2, -3, and -4 sequences (and their complementary cytosine to 3′ ends). In addition, overhang sequences 5′–CACC–3′ and 5′–AAAC–3′ were incorporated into the 5′ ends of sgRNA target sequences (and their reverse-complement sequences) to allow directional cloning into the BbsI-digested PX459 vector (Figure 2b).

Thus, in this study, four sgRNA/Cas9 expression vectors were generated: (i) PX459-sgRNA1 and PX459-sgRNA2 vectors (for exon 1) and (ii) PX459-sgRNA3 and PX459-sgRNA4 (for exon 85), as confirmed by Sanger sequencing (Figure 2c).

### 2.2. Evaluation of LRP1B Alterations in Single-Cell-Derived Clones from CRISPR/Cas9-Transfected Cells

To evaluate the ability of the developed system to silence LRP1B expression in human tumor cells, we transfected the generated vectors into the U87 glioblastoma (GB) cell line (which expresses LRP1B, contrary to the great majority of tumor cell lines [40]).

The U87 cells were transfected with PX459–empty vector (hereon referred to as “mock”) or simultaneously with (i) PX459-sgRNA1 and PX459-sgRNA2 vectors (dual targeting for exon 1), (ii) PX459-sgRNA3 and PX459-sgRNA4 (dual targeting for exon 85), and (iii) all four sgRNA/Cas9 expression vectors (dual targeting for exons 1 and 85 simultaneously). After selecting the transfected cells (with puromycin), we observed that all conditions resulted in transfected cell pools, which were subsequently diluted to isolate single transfected cell clones.

**Figure 3:**
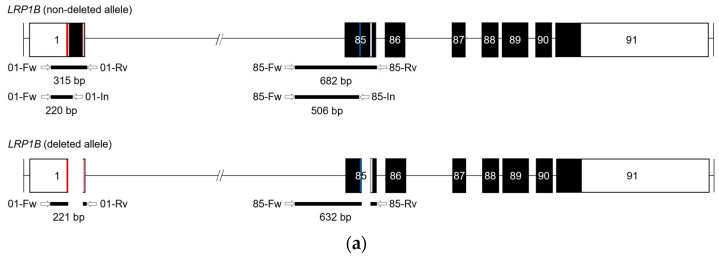

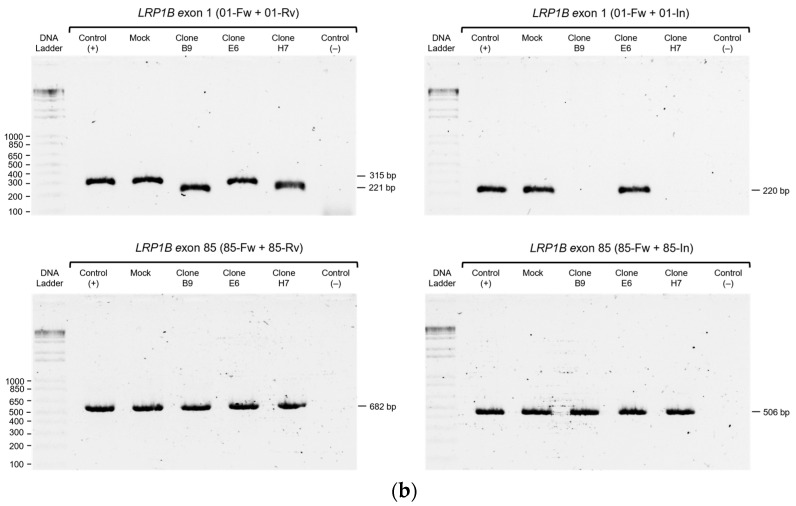
Evaluation of *LRP1B* deletions and allelic status in CRISPR/Cas9-U87 transfected clones, using conventional PCR analysis. (**a**) Schematic representation of the amplicons according to *LRP1B* allelic status. Upper panel, schematic representation of *LRP1B* non-deleted allele. Lower panel, schematic representation of LRP1B deleted allele. Boxes on the horizontal connecting black line (introns) represent exons (white: non-coding region; black: coding region). CRISPR/Cas9 target sites are shown as color vertical bars. Arrows indicate the positions and orientations of PCR primers. The expected sizes of the amplicons for *LRP1B* non-deleted and deleted alleles are also indicated (in bp). (**b**) Representative image of PCR analysis of exons 1 and 85 in three isolated clones from U87 cells transfected simultaneously with all generated PX459 vectors (sgRNA1, -2, -3, and -4). Upper panels, primers forward (01-Fw) and reverse (01-Rv or 01-In) were used for exon 1. Expected sizes of PCR amplicons for *LRP1B* non-deleted alleles are 315 bp (01-Rv) or 220 bp (01-In) and for deleted alleles are 221 bp. Lower panels, primers forward (85-Fw) and reverse (85-Rv or 85- In) were used for exon 85. Expected sizes of the amplicons for the *LRP1B* non-deleted alleles are 682 bp or 506 bp. Control (+), genomic DNA from parental (wild-type) U87 cells; negative control (−), no DNA template; 1 kbp DNA ladder.

An initial PCR screen was carried out using single-cell-derived clones to evaluate CRISPR/Cas9-mediated deletions and allelic status (as non-deleted, monoallelic-deleted, or biallelic-deleted). For this, two different PCR reactions were carried out (as depicted in Figure 3a): (i) one, using two primers located outside of the predicted deleted target region(s) (Fw and Rv), thus amplifying either non-deleted or deleted DNA fragments; (ii) the other, using one primer outside the predicted deleted exon region(s) and the other within the deleted region(s) (Fw and In). Only clones possessing at least one non-deleted allele (non-deletion or monoallelic deletion clones) would present amplification when using these primers.

PCR analysis demonstrated heterogeneity among the several clones isolated, both in terms of the presence or absence of respective amplicons, as well as differences in their size (Figure 3b; Supplementary Figures S2 and S3). Noteworthily, differences were also observed within clones isolated from the same transfection pool.

Nevertheless, our analysis of PCR results allowed us to conclude that dual sgRNA1 and sgRNA2 vectors efficiently excised exon 1 (Figure 3b upper panel and Supplementary Figure S2), while dual sgRNA-3 and -4 did not cause *LRP1B* exon 85 excision (as no alterations were observed using PCR analysis, Figure 3b lower panel and Supplementary Figure S3). Additionally, the use of both sets of dual vectors (i.e., targeting the two exons simultaneously with all four vectors) showed the same level of heterogeneity as observed with each dual strategy, with a similar ability to excise exon 1 (and not exon 85) (Figure 3).

Since PCR analysis does not allow us to fully assess the alterations induced by our CRISPR/Cas9 methodology, Sanger sequencing was carried out. Only the results obtained for three of the selected clones, herein referred to as clones B9, E6, and H7, will be presented. These were also the clones that were further characterized regarding cell phenotype. Briefly, for these specific clones, our previous PCR analysis of exon 1 indicated that (i) clones B9 and H7 were “biallelic deletion clones” due to the lack of amplification with the gene-specific primer set (01-Fw and 01-In) and that (ii) clone E6 presented a single amplicon slightly smaller than the expected sized of the non-deleted amplicon of 315 bp (Figure 3b upper panel). In addition, none of these clones showed an evident effect in exon 85-targeted regions by PCR analysis **(**Figure 3b lower panel).

**Figure 4:**
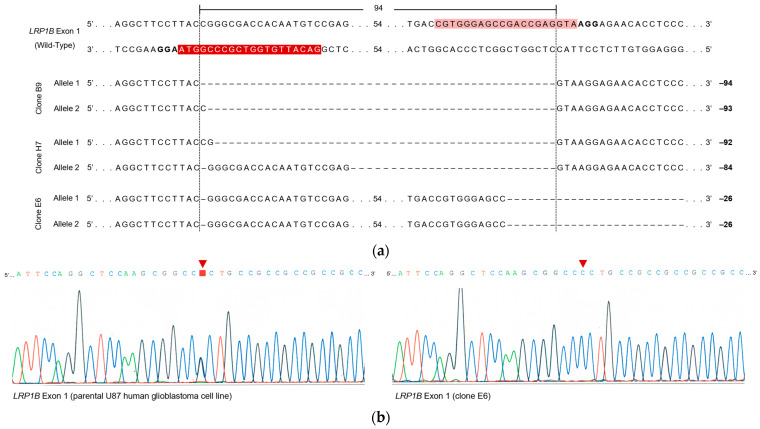

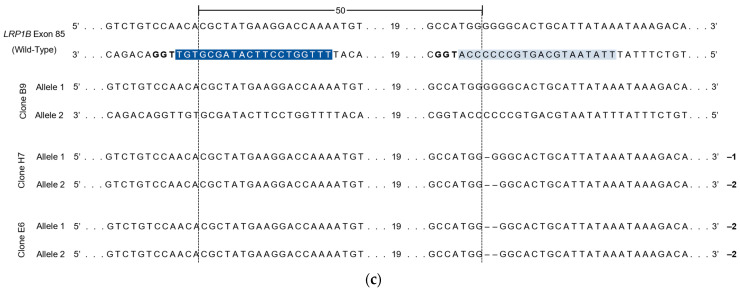
Sanger sequencing analysis of clones (B9, E6, and H7). (**a**) Amplicon sequences are aligned with the *LRP1B* wild-type sequence. PAM (bold) and DNA target (highlighted in dark and light red) sequences within *LRP1B* exon 1 are depicted in the wild-type sequence (**b**); representative electropherograms depict the presence of an SNV at the nucleotide position 210 (C → G transversion; red arrowhead) in the 5′-UTR of the human *LRP1B* in the parental U87 cell line (highlighted in red) and its absence detected in clone E6. (**c**) Amplicon sequences are aligned with the *LRP1B* wild-type sequence. PAM (bold) and DNA target (highlighted in dark and light blue) sequences within *LRP1B* exon 85 are depicted in the wild-type sequence. The number within the *LRP1B* alleles represents the number of nucleotides not shown. The vertical dashed lines indicate the predicted Cas9 cleavage site, 3 nt upstream (5′) of the PAM sequence (5′-NGG-3′). The deleted nucleotides are shown as dashes (−). The number of deleted nucleotides is displayed on the right side of the amplicon sequences (in bp).

When carrying out Sanger sequencing for exon 1 and the sequence alignment of each clone with the wild-type/parental U87 sequence (Figure 4), we observed that (i) “clone B9” harbored one allele with a precise 94 bp deletion (as mediated by accurate NHEJ) and another allele with a 93 bp deletion (Figure 4a); (ii) ” clone H7 “ harbored two alleles, one with 92 bp and the other with a 84 bp “deletion”, which included insertions with partial homology to the sequence within the predicted CRISPR/Cas9-mediated deletion (Figure 4a); and (iii) “clone E6” had a 1 bp deletion in the sgRNA1-binding region and another 25 bp deletion in the sgRNA2-binding region (Figure 4a). Additionally, clone E6 did not have a single-nucleotide variant (SNV) at the nucleotide position 210 (C → G transversion) in the 5′-UTR of the human *LRP1B* (which is upstream the target region within LRP1B exon 1), in contrast to the *wt* U87 cells (Figure 4b, red arrowhead).

Upon analyzing the exon 85 of the selected clones using Sanger sequencing, although no large alterations were expected since no deletions were verified by PCR (Figure 3b), small deletions were found in clones E6 and H7 (Figure 4c). These were 1 bp to 2 bp deletions in the sgRNA4-binding region (light blue, Figure 4c). For clone B9, no deletions were found.

An outline of *LRP1B* alterations observed in the selected clones, along with their predicted effect on *LRP1B* mRNA (which will be further discussed in the subsequent sections), is depicted in Table 1.

### 2.3. Evaluation of LRP1B Silencing in Cell Clones

**Figure 5:**
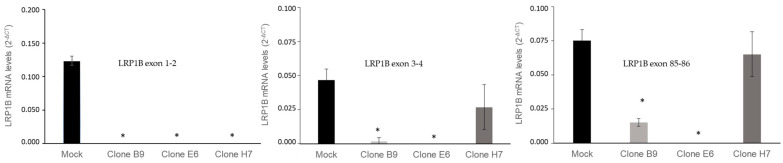
*LRP1B* expression in U87 mock cells and clones B9, E6, and H7. mRNA *LRP1B* levels were evaluated with RT-qPCR using TBP as the endogenous control. Analysis was carried out with primer assays for the following *LRP1B* mRNA regions: exons 1-2 junctions; exons 3-4 junctions; and exons 85-86 junctions. Results are the mean ± SEM of at least three independent experiments. * *p* < 0.05.

To assess if our CRISPR/Cas9 system was able to generate LRP1B knockout U87 cells, we evaluated *LRP1B* expression using qRT-PCR (Figure 5) in all clones and further confirmed decreased LRP1B protein expression using immunofluorescence in clones E6 and H7 (Supplementary Figure S4). Using a primer assay for *LRP1B* exon 1-2, the abolishment of *LRP1B* expression was observed in all three selected clones (Figure 5), indicating the success of our methodology. Still, as CRISPR/Cas9 systems may not result in the total elimination of the gene-targeted regions (as previously observed in this study) and may also result in unexpected alterations in mRNA expression [44], we evaluated other *LRP1B* mRNA regions, using specific real-time primer assays for *LRP1B* exons 3-4 and 85-86 junctions.

The results further confirmed the abolishment of all *LRP1B* mRNA transcripts analyzed in clone E6 (Figure 5). These results indicate that clone E6 (which had a 25 bp deletion of the *LRP1B* exon 1–intron 1 junction) resulted in the complete abrogation of *LRP1B* mRNA (no amplification in exon 1-2, 3-4, and 85-86 transcripts was observed). In clones B9 and H7, although none showed mRNA expression of exon 1-2, *LRP1B* exon 3-4 and exon 85-86-associated transcripts were still detected, although decreased. Although it is expected that the presence of these abnormal transcripts will not lead to the translation of the full LRP1B gene [45], as confirmed by immunofluorescence (Supplementary Figure S4), we should not disregard their presence and potential effect on cell behavior.

**Figure 6:**
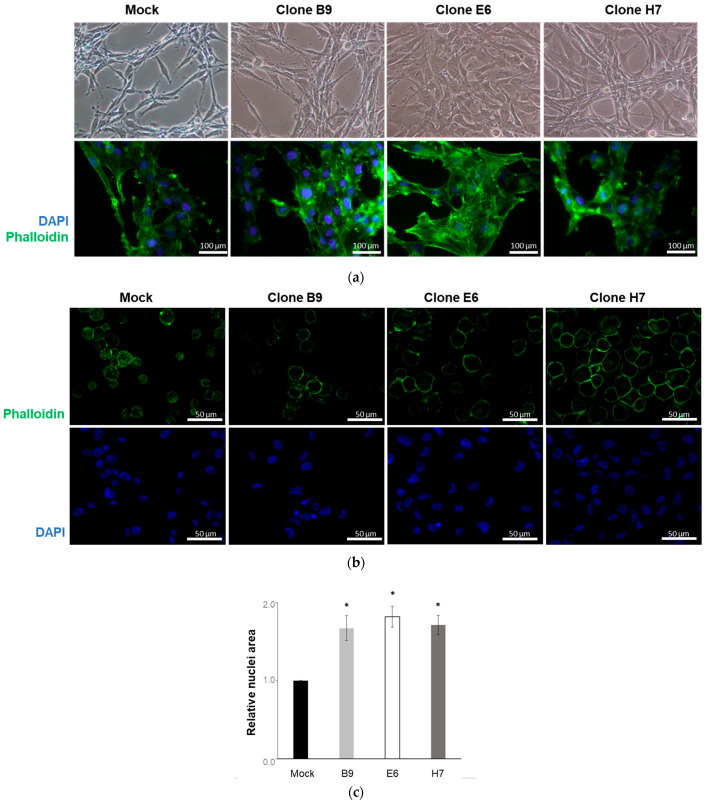
U87 LRP1B-edited clones presented altered morphology compared to mock cells. U87 mock cells and clones B9, E6, and H7 were evaluated for (**a**) cell morphology in adherent cells in culture using phase contrast microscopy (upper panel) or fluorescence microscopy (lower panel). (**b**) Cell and nuclei size. Representative images of cells after cytospin, showing increased cell/nuclear size in silenced clones. All fluorescent microscopy images are of actin staining with phalloidin (green) and nuclear staining with DAPI. (**c**) Cell nuclei area (evaluated with ImageJ software version 1.5.1with NII plugin) analyzed in relation to mock cell nuclei area. Results are the mean ± SEM of three independent experiments. * *p* < 0.05 vs mock. Note that wild-type cell morphology and cell nuclei area were similar to mock cells (Supplementary Figure S5).

We next evaluated eventual alterations in the morphology of the selected CRISPR/Cas9-altered clones. Our results showed that the established *LRP1B* gene alterations have a clear impact on U87 cell morphology (Figure 6a). While the U87 mock cells presented the expected parental cell phenotype resembling neuronal morphology with long thin protrusion features [46] (Supplementary Figure S5), the other three clones presented a more flattened, epithelial phenotype, with increased cell contact (particularly evident in clone E6). Using immunofluorescence microscopy, the cell cytoskeleton was observed after actin staining with phalloidin. In all the selected clones, and again more evidently in clone E6, LRP1B alterations affected the cell cytoskeleton with an increase in the presence of actin fibers and augmented the cells’ overall size (Figure 6a).

**Figure 7:**
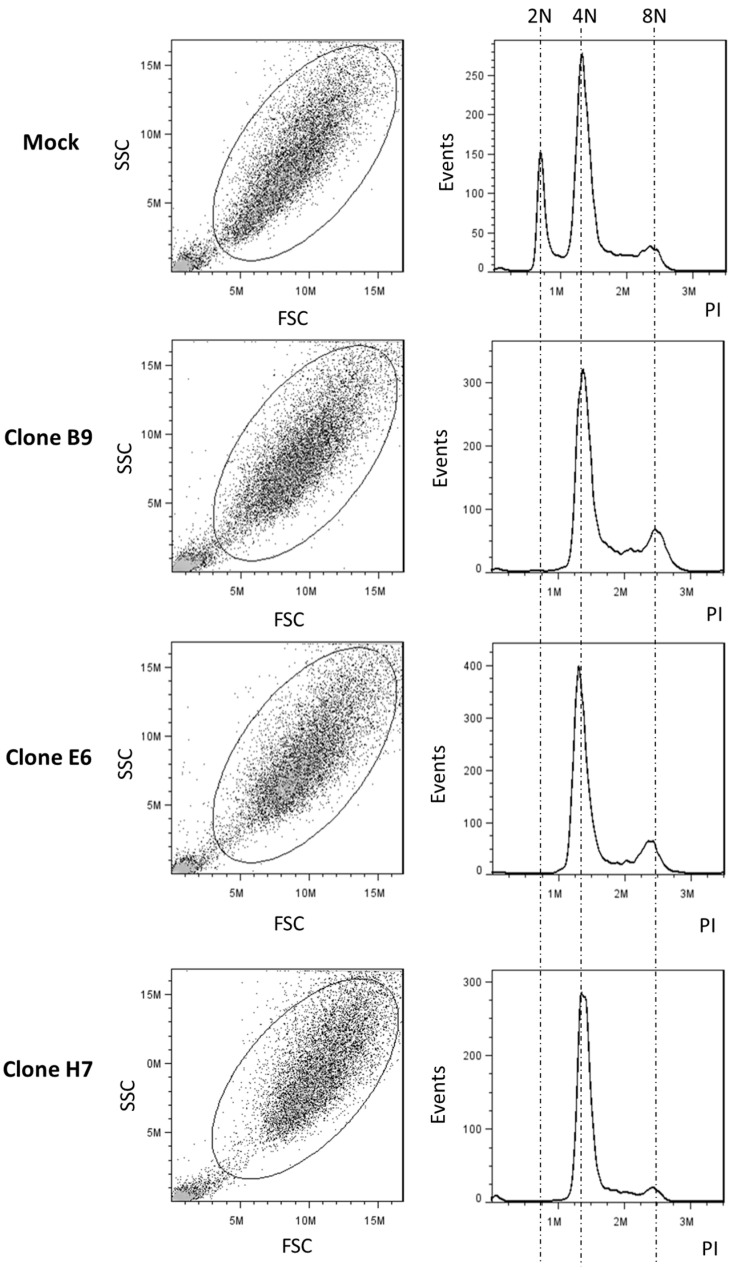
Flow cytometry analysis of LRP1B-edited U87 cell clones. Left panels correspond to dot plots of side vs. forward scatter (SSC vs. FSC) and show the gated population. An increase in cell size and complexity is observed in the three evaluated clones compared to control cells. Right panels correspond to the evaluation of the cell cycle profile of the gated population (DNA staining with propidium iodide (PI)), following the exclusion of cellular aggregate debris and doublets. Histograms show the absence of a near-diploid population and an aneuploidy/polyploidy phenotype (i.e., cells with DNA content > 4N or > 8N) in the three clones when compared to mock cells. The images are representative of three independent experiments. Cell cycle profile analysis of *wt* cells is shown in Supplementary Figure S5.

A clear increase in nuclear size was observed in all the three studied clones when compared to the mock (and *wt*) cells (Figure 6b,c; Supplementary Figure S5). The increased complexity and cell size of the three clones (compared to *wt* and mock cells) were further verified with flow cytometry analysis (side scatter (SSC) vs. forward scatter (FSC) analysis, Figure 7 and Supplementary Figure S5).

Moreover, since a correlation between nuclear volume and genome size has also been described in other studies [47], we hypothesized that the nuclear enlargement of the three clones could be associated with increased DNA content [48]. We thus evaluated the DNA content and cell cycle profile of the U87 clones using flow cytometry analysis after propidium iodide staining. The results of the three clones showed that they all shared an identical cell cycle profile and were considerably different from the mock and *wt* cells (Figure 7 and Supplementary Figure S5), compatible with a progression from a diploid to a polyploidy/aneuploidy state.

### 2.4. Effect of LRP1B Alterations on Tumor Growth In Vitro and In Vivo

**Figure 8:**
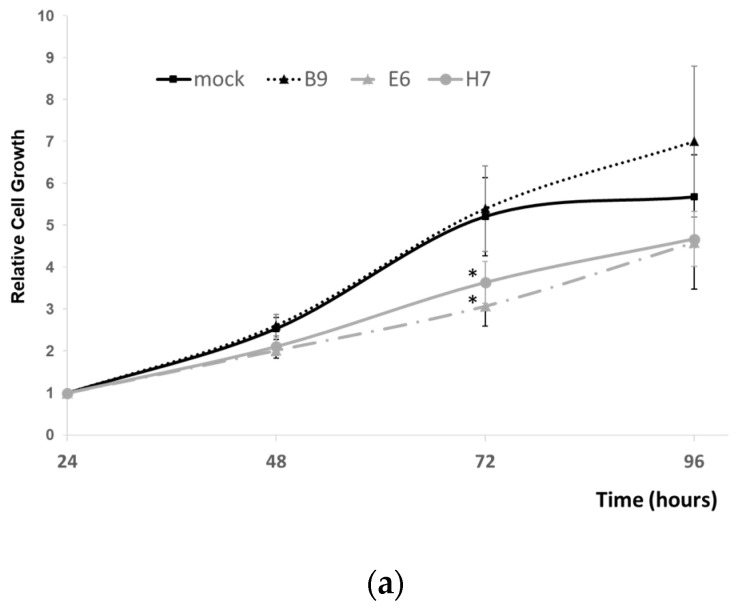

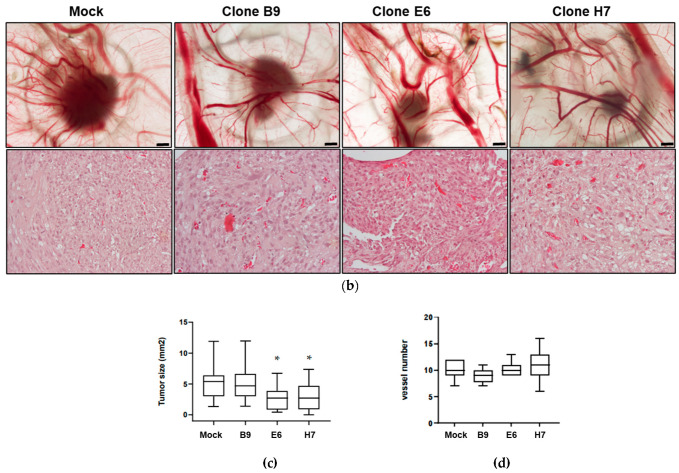
Evaluation of the growth of U87 mock cells and selected clones (B9, E6, and H7) in vitro and in vivo. (**a**) Cell growth analysis in vitro, with Presto blue viability assay following 24 h, 48, 72, and 96 h in culture. Results, expressed in relation to the 24 h, are the mean ± SEM of at least three independent experiments. * *p* ≤ 0.05 vs mock cells. (**b)** Upper panel—representative “ex ovo” images of CAM xenografted tumors. It is possible to observe the mark of the ring used for cell inoculation, as well as the tumors and newly formed blood vessels. Bar = 500 µm. Lower panel—tumor histology evaluated by H&E staining (200X magnification). (**c**) Tumor growth analysis in vivo: CAM xenografted tumor areas measured in mm^2^. Results are mean ± SEM from independent experiments (total number of technical replicates: mock, n = 11; clone B9, n = 12; clone E6, n = 10; and clone H7 = 10); * *p* < 0.05 vs. mock cells. (**d**) Angiogenic assay. Quantification of the number of vessels growing towards the inoculation site (total number of technical replicates: mock, n = 8; clone B9, n = 3; clone E6, n = 7; and clone H7 = 5). * *p* < 0.05 vs. mock cells.

To further evaluate how the alterations carried out on the *LRP1B* gene affected U87 cell growth, the PrestoBlue viability assay was used. Our results showed that clones E6 and H7 had a significantly decreased growth compared to controls (and *wt* cells, Supplementary Figure S5), which was not observed for clone B9 (Figure 8a). Most importantly, this impact of *LRP1B* alterations was further confirmed in vivo by evaluating the ability of each selected clone to grow and form tumors in the chick embryo chorioallantoic membrane—CAM (Figure 8b upper panel and Figure 8c). The results in this in vivo model were consistent with the ones previously obtained in vitro *(*Figure 8a), where a significant reduction in tumor growth was observed for clone E6 and H7 xenografts in comparison to the mockxenografts (while no differences for clone B9 were observed) (Figure 8c). In addition, it was possible to histologically confirm differences in their phenotype when compared to the mock. The tumors formed by LRP1B-edited clones presented increased nuclear size, and clone E6, in particular, presented increased cell contact (Figure 8b upper panel). Taking advantage of the potential of the CAM assay, we also evaluated angiogenesis by comparing the number of new vessels induced by xenografted cells (Figure 8d). No significant alterations were observed in the angiogenic response of the tested cells.

### 2.5. Effect of LRP1B Alterations on Cell Secretome

**Figure 9:**
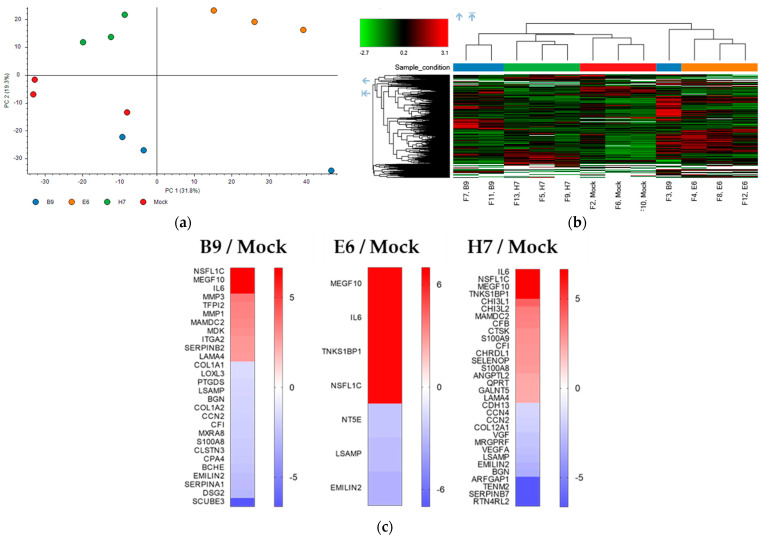
Proteomic analysis of secretomes derived from U87 mock cells and clones B9, E6, and H7. (**a**) Principal component analysis (PCA) and (**b**) hierarchical clustering of each sample, showing a clear separation of the proteomic profile of all conditions. (**c**) Heatmaps of DEPs in each of the clones (B9, E6, or H7) versus mock-derived secretomes. Results are from three independent experiments, after excluding contaminants. Only proteins with an adjusted *p*-value of <0.05 and a number of unique peptides > 2 were included.

Considering that LRP1B has previously been characterized as a scavenger receptor [49] and a modulator of cell secretome following its overexpression in tumor cells [12], we aimed to obtain a more profound comprehension of the consequences of LRP1B deletion in GB cells on extracellular protein expression (secretome) and thus its potential impact on the tumor microenvironment. To accomplish this, a global proteomic analysis of the conditioned medium of all clones was performed using mass-spectrometry-based proteomics. Three biological replicates of each cell-derived secretome (U87 mock and B9, E6, and H7 clones) were profiled using LC-MS with subsequent bioinformatic analysis. Our proteomic analysis identified a total of 2230 proteins present in the secretomes of all samples. Principal component analysis (PCA) revealed a global separation of the proteomic profile of all conditions, especially when comparing mock cell secretomes with clone E6 and H7 cell-derived secretomes (Figure 9a). This same trend was evident from the hierarchical clustering (Figure 9b), suggesting that the secretomes derived from LRP1B-silenced clones clearly diverge in their proteome profile from mock-cell-derived secretomes.

To identify proteins that were differentially expressed (DEPs) in the secretome of mock cells and each clone-derived secretome, we applied three filters to the total list of proteins identified using LC-MS. These filters included removing common contaminants, the selection of proteins with more than two unique peptides, and FDR-adjusted *p*-values below 0.05 (the filtered list of proteins can be found in Supplemental Data Excel Files S1–S6). Using the Proteome Discoverer software (version 2.5.0.400), 1088 proteins were identified across all conditions and were displayed as heatmaps highlighting the DEPs in each clone versus the mock-derived secretomes (Figure 9c).

In the comparison of secretomes from LRP1B-altered clones with secretomes from mock cells, (i) clone B9 had 28 DEPs, with 11 being significantly upregulated and 17 downregulated; (ii) clone E6 showed 7 DEPs, with 4 being significantly upregulated and 3 downregulated; and (iii) clone H7 had 32 DEPs, with 18 being significantly upregulated and 14 downregulated. Multiple Epidermal Growth Factor-like Domains 10 (MEGF10), Interleukin-6 (IL-6), and NSFL1 cofactor p47 (NSFL1C) were significantly upregulated, while Limbic-System-Associated Membrane Protein (LSAMP) and Elastin Microfibril Interfacer 2 (EMILIN-2) were significantly downregulated, in all clone-derived secretomes compared with mock secretomes (Table S5). None of these proteins have been previously described to be associated with LRP1B.

## 3. Discussion

Despite considerable research, the precise role of LRP1B in cancer has yet to be fully elucidated. However, recent advances in gene editing technologies, such as CRISPR/Cas9, have provided a new opportunity to manipulate LRP1B gene and protein expression in cells, which may enable the investigation of LRP1B’s functional role in cancer.

Here, we explored the potential of CRISPR/Cas9-mediated gene editing to silence LRP1B in cancer cells to gain a deeper insight into the functional role of LRP1B. We used the U87 GB cell line as a model. Genomic *LRP1B* losses were proposed as candidate genomic markers related to progression to glioblastoma in primary glioma cell lines [50]. In addition, *LRP1B* deletions have been associated with poor overall survival in GB patients [11]. Still, the impact of *LRP1B* genetic alterations (as well as its mRNA and protein levels) remains unclear. Therefore, creating *LRP1B*-deleted clones of one of the most used GB cell lines could offer a valuable insight into the role of LRP1B in this setting.

Our strategy to silence the *LRP1B* gene was to employ a dual sgRNA CRISPR/Cas9 deletion approach (using two sgRNAs to guide the Cas9 activity to sites flanking one target region), which has been successfully used in the deletion of protein-coding genes [51,52,53], non-coding RNA (sncRNA) [54,55], long non-coding RNA (lncRNA) [28,51], and enhancers [56]. Furthermore, to enhance our silencing strategy, we generated four sgRNA-CRISPR/Cas9 vectors to target two *LRP1B* exons, namely exon 1 and exon 85, either individually or simultaneously.

Overall, we were able to alter the *LRP1B* gene in U87 transfected cells. While dual sgRNA1 and sgRNA2 vectors efficiently excised exon 1 (Figure 3 and Supplementary Figure S2), dual sgRNA3 and sgRNA4 vectors failed to cause *LRP1B* exon 85 excision (Figure 3 and Supplementary Figure S3). This was likely due to the low *on-target* score of the sgRNA3 (32.1/100) and of sgRNA4 (44.2/100), indicative of the reduced efficiency of sgRNA binding and Cas9 cleavage at the target site [57] (Supplementary Table S1). The results obtained with the simultaneous use of all four vectors showed a similar ability to excise exon 1 (and not exon 85) (Figure 3).

Our study using PCR and Sanger sequencing analysis revealed a noteworthy level of heterogeneity in the *LRP1B* alterations across various U87 transfected cell clones, including those isolated from the same pool of transfected cells. This could be potentially attributed to the dual approach used in this study, along with the simultaneous targeting of two exons, increasing the likelihood of unintended effects [29]. Nonetheless, our results are consistent with previous work showing that the efficacy and precision of CRISPR/Cas9 depend on multiple factors, such as the target genome, Cas9 concentration, delivery method, and specific characteristics of the cells being studied [58,59]. The heterogeneity found in our study regarding *LRP1B* gene alterations among clones emphasizes the importance of carefully evaluating gene editing following CRISPR/Cas9 before proceeding with further analysis. Specific genetic differences may have varying effects on gene function and subsequent cellular phenotype. This holds particular relevance for genes such as *LRP1B*, whose deletion/mutations, although not fully understood, have been associated with cancer patients’ prognosis and response to therapy [6].

Therefore, in this study, we further investigated three of the *LRP1B*-altered clones, namely clones B9, E6, and H7, which were all derived from the same U87 cell transfection condition, targeting both exons and thus potentially having more impact on *LRP1B.*

In particular, the sequencing of exon 1 showed that clones B9 and H7 were more similar (Figure 4), although (i) clone B9 had one allele with a precise 94 bp deletion (as mediated by accurate NHEJ) and another with a 93 bp deletion and (ii) clone H7 had two alleles, one with a 92 bp and the other with 84 bp “deletion” which included insertions with partial homology to the sequence within the predicted CRISPR/Cas9-mediated deletion. These CRISPR/Cas9-mediated deletions caused (i) the truncation of the 5′ UTR (in which 10 nt to 12 nt at the 3′ end is deleted) and (ii) the loss of the canonical AUG start codon on *LRP1B* mRNA.

The evaluation of clone E6 exon 1 showed a 1 bp deletion in the sgRNA1-binding region and another 25 bp deletion in the sgRNA2-binding region (Figure 4). The 25 bp deletion in the sgRNA2-binding region may have resulted in the elimination of the last 7 bp of *LRP1B* exon 1 and the first 18 bp of *LRP1B* intron 1, disrupting the 5′ splice site and potentially leading to exon skipping, intron retention, or the introduction of a new splice site within an exon or intron [60]. Guo et al. [61] indicated that the frequency of accurate NHEJ in repairing two (close and concurrent) Cas9-induced DSBs was hampered by frequent 1 bp and 2 bp insertions at the predicted deletion junctions. In fact, several studies have shown that Cas9 generates predominantly blunt DNA ends but occasionally DNA ends with 5′ overhangs, particularly 1 nt and 2 nt 5′ overhangs that often result in template-dependent insertions [62,63,64,65,66].

When evaluating exon 85, we also found small deletions (1 bp to 2 bp) in the sgRNA4-binding region for clones E6 and H7 (but not in clone B9). Although our results showed that the SpCas9, together with the pair sgRNA3 and sgRNA4, was unable to excise the targeted region of *LRP1B* exon 85, the sgRNA4/Cas9 complex seems to be able to bind to the DNA target sequence and induce a DSB within the target DNA (3 nt upstream (5′) of the PAM sequence; Figure 3). This agrees with the fact that the on-target score for sgRNA4 (44.2 out of 100) was higher than the one for sgRNA3 (32.1 out of 100) (Supplementary Table S1).

To assess the impact of the genetic modifications in *LRP1B*, we analyzed the expression of different transcript regions. Our results confirmed that all *LRP1B* mRNA transcripts were lost in clone E6 (Figure 5), indicating that the 25 bp deletion of *LRP1B* exon 1–intron 1 junction resulted in the abrogation of LRP1B mRNA. The disruption of a splice site can result in exon skipping, intron retention, or in the introduction of a new splice site within an exon or intron [60]. We hypothesized that the complete ablation of the *LRP1B* mRNA might be attributed to intron 1 retention and the consequent establishment of a premature termination codon (PTC), eliciting nonsense-mediated decay (NMD) of the mutant *LRP1B* mRNA. Intron retention has been associated with the downregulation of gene expression via the NMD pathway [67] as retained introns often interrupt the main open reading frame (ORF) of the mRNA and lead to the introduction of PTCs [68]. Regarding clones B9 and H7, although *LRP1B* mRNA expression for exon 1-2 was not detected, the presence of *LRP1B* exons 3-4 and 85-86-associated transcripts was observed, albeit at decreased levels (Figure 5). It was expected that the translation of altered mRNA would be entirely abrogated (due to the canonical AUG start codon’s removal); however, it might still have proceeded from a downstream alternative start codon (the first of which is found at *LRP1B* exon 2). Although these transcripts would not lead to the translation of the full *LRP1B* gene, we cannot exclude the possible translation of alternative transcripts nor their potential role in cell biology.

Therefore, we further proceeded to assess the phenotype of the LRP1B-altered clones and compared them to mock cells, which retained a similar phenotype to wild-type cells. All LRP1B-altered clones exhibited morphological changes characterized by a more flattened, epithelial phenotype and increased cell contact, with the most notable changes observed in clone E6 (Figure 6). In contrast, U87 mock cells maintained a normal, neuronal morphology with long, thin protrusions [46]. Immunofluorescence microscopy revealed an increase in the presence of actin fibers and overall cell size in all LRP1B-altered clones, with the most significant changes again observed in clone E6. The remodeling of the actin cytoskeleton and alteration of focal adhesion complex components have previously been shown in LRP1B-silenced cells, although in that context they were associated with increased migration and invasion [18].

All LRP1B-altered clones exhibited an increase in nuclear and cell size, associated with an increase in DNA content, when compared to mock and wild-type cells (Figure 6 and Figure 7 and Supplementary Figure S5). It is worth noting that the U87 cell line has a slightly hypodiploid karyotype, with 43 to 45 chromosomes [69], and has been reported to have a high cytogenetically aberrant profile with an increased rate of higher ploidy cells of almost 6% [70]. Transfection itself increased the higher ploidy cell population, as evidenced by the comparison of cell cycle profiles between mock and wild-type cells in our study (Supplementary Figure S5). Previous studies have also reported an increase in genetic instability and ploidy in U87 cells after transfection (with erythropoietin receptor shRNA vectors [71]). However, our study revealed a dramatic change in the cell cycle profile in LRP1B-altered clones, with a loss of diploid cells (2N) and a shift towards tetraploid (4N) and higher ploidy cell populations. While it is possible that in our LRP1B genetically modified clones there was an overgrowth of pre-existing U87 polyploid cells that may have replaced the near diploid cells, our results suggest that *LRP1B* deletions are involved in increased ploidy, which is highly associated with genomic instability [72,73]. Indeed, previous studies have associated *LRP1B* deletions and mutations with high TMB in several types of cancer, including melanoma, lung, and hepatic cancer, reflecting a high genomic instability [10]. The association between the loss of *LRP1B* and genetic instability (high tumor mutation burden) has been noted in several types of cancers [10] and has been linked to a high response rate to immune checkpoint inhibitors, resulting in favorable patient outcomes [22,25]. However, the potential link between *LRP1B* genetic alterations and tumor mutation burden/tumor instability needs to be further addressed in other studies [22].

The effects of LRP1B silencing, or of polyploidy itself, on tumor cell growth are still debatable and may be dependent on the cellular context. While some studies have reported that LRP1B silencing with siRNA/shRNA results in increased proliferation, migration, and invasion in several cancer cell lines [14,18,19], others have shown that lentiviral CRISPR/Cas9 LRP1B knockdown inhibited cell growth in HCC cells [20]. Interestingly, these cells also overexpress LRP1B, contrary to most of the established tumor cell lines [20].

In our study, we further evaluated the effect of LRP1B silencing on tumor growth. We found that two of the three clones (E6 and H7) showed a significantly decreased growth compared to mock cells, while clone B9 did not show a significant difference (Figure 8). These results were validated both in vitro and in vivo using the CAM assay (Figure 8). Further corroborating the in vitro findings, CAM xenografted tumors from LRP1B-edited clones showed different morphologies compared to mock cells, with increased cell contact and nuclear size (which was more evident in clone E6). The angiogenic response of transfected cells (mock and LRP1B-altered clones) was also quantified using the CAM assay. The results showed no significant differences in the number of vessels among the groups, suggesting that the loss of LRP1B might not be directly involved in the angiogenic potential of U87 cells (Figure 8). However, further studies are required to confirm this finding as in different cell line models and following LRP1B (minireceptor) overexpression, an association with angiogenesis has been observed [12].

Finally, and given that LRP1B was identified as a secretome modulator in thyroid cancer cells [6,7,12], we investigated the differences in the secretomes of the three GB clones. Our analysis revealed a relatively low number of differentially expressed proteins (DEPS) that were common to all of the LRP1B-silenced clones. These included MEGF10, IL6, and NSFL1C, which were significantly upregulated, and LSAMP and EMILIN2, which were significantly downregulated in all clone-derived secretomes compared to mock secretomes. Although this suggests that these proteins may play a role in LRP1B function, to the best of our knowledge there is no information on a direct association between LRP1B and any of these molecules. Regarding the roles described for the identified DEPs (or pathways in which these interfere), there are some potential links that may be explored in future studies. For example, MEGF10 acts as a scavenger receptor. Albeit belonging to a different receptor class from LRP1B, both share identical motifs [49]. Specifically, MEGF10 participates in the uptake of amyloid-β peptide, which is also a ligand for LRP1B in the brain [6,74]. IL-6 has been previously associated to LRP1, which is highly homologous of LRP1B (and its most closely related LDLR family member). Increased IL-6 synthesis was observed in LRP1 knockout macrophages [75] and, in microvascular endothelial cells, IL-6 with other pro-inflammatory cytokines mediated the downregulation of LRP1 [76]. Furthermore, there is evidence that IL-6 may also promote polyploidization [77]. The collected data regarding the differentially expressed proteins (DEPs) following LRP1B silencing, along with their associated pathways, offer new insights into the functional role of LRP1B in cancer cells. To further conduct a more comprehensive analysis of the association between LRP1B absence and the observed cellular alterations, it is expected that the evaluation of the cellular proteome in our established models will yield valuable information. This assessment will provide additional validation of our findings, contribute to a deeper understanding of the impact of LRP1B’s role, and establish the clinical relevance and translational potential of our research.

## 4. Materials and Methods

### 4.1. LRP1B Target Region

The human *LRP1B* gene sequence (Ensembl ID: ENS00000168702, accessed on 2 September 2019), in particular exons 1 and 85, was chosen as the targets. Benchling’s CRISPR Guide RNA Design Software (https://www.benchling.com/crispr/, accessed on 2 September 2019) was used to identify 5′-NGG-3′ PAM sequences within target regions and list all possible sgRNA target sequences. Selection of gRNA target sequences (Table S1) was based on their predicted target specificity (off-target score [37] and cleavage efficiency (on-target score) [57]). Potential off-target sequences within the human genome (GRCh38; [78]) were identified using the same software (Supplementary Table S2).

### 4.2. CRISPR/Cas9 Expression Vector

pSpCas9(BB)-2A-Puro (PX459) V2.0, a gift from Feng Zhang (Addgene plasmid # 62988; http://n2t.net/addgene:62988;RRID:Addgene_62988 accessed on 3 June 2023), was used as the backbone for the construction of all sgRNA/Cas9 expression vectors used in this study [38]. The PX459-containing *Escherichia coli* Stbl3 strain (supplied as a stab culture) was handled according to the provider’s recommendations (Addgene, Watertown, MA, USA), and plasmid DNA was recovered using the NZYMidiprep Kit (NZYTech, Lisbon, Portugal) following the manufacturer’s instructions. Before sgRNA cloning, plasmid DNA (5 μg) was digested with 25 U of BbsI in the presence of 1 × buffer G (with bovine serum albumin (BSA)) for 6 h at 37 °C (both from Thermo Fisher Scientific, Waltham, MA, USA), separated by electrophoresis on a 0.8% (*w*/*v*) agarose gel, and further isolated with Cut and Spin Gel Extraction Columns (GRiSP, Porto, Portugal) according to the manufacturer’s instructions.

### 4.3. sgRNA Oligonucleotide Duplex Preparation and Cloning into PX459 Vector

Oligonucleotide pairs used for sgRNA cloning are listed in Supplementary Table S3. Prior to sgRNA cloning, oligonucleotide pairs (10 μM each; Integrated DNA Technologies, Leuven, Belgium) were phosphorylated and annealed using 1.5 U T4 polynucleotide kinase (NZYTech, Lisbon, Portugal) as follows: 37 °C for 30 min, 95 °C for 5 min, and then cooled to 25 °C at a rate of 1 °C/min. Ligation of each sgRNA-encoding oligonucleotide duplex (1 μL) into BbsI-digested PX459 vector (50 ng) was carried with 5 U T4 DNA ligase (Thermo Fisher Scientific, Waltham, MA, USA) for 1 h at 22 °C. Ligation products were transformed into chemically competent *E. coli* DH5α cells and plated onto LB–ampicillin agar plates [79]. After overnight incubation at 37 °C, colonies were randomly picked and screened for correct insertion of sgRNA target sequences. All generated vectors were confirmed using Sanger sequencing (at i3S Genomics Platform, i3S, Porto, Portugal) using U6 promoter forward primer: 5′–GAGGGCCTATTTCCCATGATTCC–3′.

### 4.4. Cell Culture 

U87 human glioblastoma cell line (a kind gift from Dr. Bruno Costa, ICVS, School of Medicine, University of Minho, Braga, Portugal) was used. Cells were genotyped at i3S Genomics Core Facility (Porto, Portugal) using the PowerPlex^®^ 16 HS System (Promega, Madison, WI, USA) for authentication based on DNA profiles available on the ATCC and ECACC STR profiles database [16] and evaluated for mycoplasma contamination using PCR. Cells were routinely maintained in Dulbecco’s Modified Eagle Medium (DMEM) high glucose (4.5 g/L) with stable glutamine and sodium pyruvate (Capricorn Scientific, Hessen, Germany) supplemented with 10% (*v*/*v*) Gibco™ heat-inactivated fetal bovine serum (FBS; Thermo Fisher Scientific, Waltham, MA, USA), 1% (*v*/*v*) penicillin–streptomycin (Biowest, Nuaille, France), and 0.5% (*v*/*v*) amphotericin B (Thermo Fisher Scientific, Waltham, MA, USA) at 37 °C in a humidified atmosphere with 5% CO_2_.

### 4.5. Cell Transfection

Cells transfection with CRISPR/Cas9 expression vectors was carried out using the cationic-lipid-based Lipofectamine™ 3000 transfection reagent (Thermo Fisher Scientific, Waltham, MA, USA) according to the manufacturer’s protocol. Briefly, 7.5 × 10^4^ cells/well were plated in a 24-well plate for 24 h to allow adhesion and then co-transfected with two or four sgRNA/Cas9 expression vectors (500 ng and/or 1000 ng for the transfection with four vectors) in OPTIMEM. Following 6 h transfection, the medium was replaced, and cells were incubated for 24 h before selection with 1 μg/mL puromycin for 3 days. Upon antibiotic removal (72 h post-selection), transfected cell pools were further diluted to obtain single-cell clones. Cells were observed daily using optical microscopy to evaluate the establishment of single-cell clones. These clones were scaled up and further processed according to the following protocols.

### 4.6. Genomic DNA Extraction, PCR, and Sanger Sequencing Analysis

Genomic DNA (gDNA) was extracted from cell pellets using the GRS Genomic DNA Kit—Cultured Cells (GRiSP, Porto, Portugal), according to the manufacturer’s instructions. CRISPR/Cas9-mediated genomic deletions were screened with PCR using gDNA as the template and specific oligonucleotide primers (Supplementary Table S4; [80]). Each PCR reaction (10 μL) contained 50 ng of gDNA, 0.25 μM of each primer, and 1 × MyTaq™ HS mix (Bioline) and was performed as follows: 1 cycle at 95 °C for 2 min, 10 cycles at 95 °C for 30 s, 68−58 °C for 30 s (−1 °C per cycle), 72 °C for 30 s, 30 cycles at 95 °C for 30 s, 58 °C for 30 s, 72 °C for 30 s, and finally 1 cycle at 72 °C for 1 min. PCR products (amplicons) were resolved through 1.0% (*w*/*v*) agarose gels, and their sizes were estimated by comparison with 1 Kb plus DNA ladder (Thermo Fisher Scientific, Waltham, MA, USA). Then, CRISPR/Cas9-mediated genomic deletions were further characterized by Sanger sequencing. Amplicons (7.5 μL) were subjected to a post-reaction clean-up with 10 U of exonuclease I and 1 U of FastAP™ thermosensitive alkaline phosphatase (both from Thermo Fisher Scientific, Waltham, MA, USA) for 30 min at 37 °C. After enzyme inactivation (85 °C for 15 min), cycle sequencing reactions were performed using BigDye™ Terminator v3.1 Cycle Sequencing Kit (Thermo Fisher Scientific, Waltham, MA, USA). Each reaction contained 0.50 μL of purified PCR amplicon, 0.25 μL of the selected PCR primer (10 μM), 0.25 μL of BigDye™ terminator, 3.50 μL of sequencing buffer (5×), and 3.50 μL of nuclease-free distilled water and was conducted as follows: 95 °C for 2 min, 35 cycles at 95 °C for 15 s, 55 °C for 15 s, 60 °C for 2 min, and 10 min at 60 °C. After the purification of the sequencing reactions by gel filtration through Sephadex™ G−50 fine (GE Healthcare, Chicago, IL, USA) spin columns (1100 g, 4 min, RT), these were mixed with 15 μL Hi-Di™ formamide (Thermo Fisher Scientific, Waltham, MA, USA) and subjected to capillary electrophoresis on a 3130/3130xl Genetic Analyzer (Thermo Fisher Scientific, Waltham, MA, USA) at the i3S GenCore Facility (Oporto, Portugal).

### 4.7. RNA Extraction and LRP1B mRNA Expression Analysis

Total RNA was extracted from cell pellets using TripleXtractor (GRiSP, Porto, Portugal) according to the manufacturer’s instructions. DNase-treated RNA (500 ng or1 μg) was reverse-transcribed using 100 μM random hexamer primers (5 × reaction buffer, 20 U RiboLock™ RNase inhibitor, 20 mM dNTP mix, and 200 U RevertAid™ reverse transcriptase). “-RT” (“minus reverse transcriptase enzyme”) reactions were included. Reverse transcription reactions were carried out as follows: 10 min at 25 °C, 60 min at 42 °C, and 10 min at 70 °C. Real-time PCR analysis was carried out using TaqMan Universal PCR Master Mix (Thermo Fisher Scientific, Waltham, MA, USA) with the following primer assays (IDT, Leuven, Belgium): Hs.PT.58.21358572 (*LRP1B* exon 1-2), Hs.PT.58.19387941 (*LRP1B* exon 3-4), Hs.PT.58.14536045 (*LRP1B* exon 85-86), and Hs.PT.39a.22214825 (TBP) as housekeeping control under the following conditions: 95 °C for 5 min; 40 cycles at 95 °C for 15 s, followed by 60 °C for 40 s. Each sample (from at least 3 independent experiments) was run in triplicate in a QuantStudio5 Real-Time PCR System (Applied Biosystems, Foster, CA, USA). All experiments included non-template (without cDNA) and “-RT” controls. LRP1B mRNA levels were analyzed using the 2^−ΔCT^ method (in which *CT* stands for “cycle threshold” and Δ*CT* = *CT* [*LRP1B*] − *CT* [*TBP*]).

### 4.8. Cell Growth and Cell Cycle Profile Analysis

Cells (5 × 10^3^ cells/well) were plated on 96-well plates in supplemented media and incubated for 96 h. At 24h, 48 h, 72 h, and 96 h, PrestoBlue™ cell viability assay was carried out, as previously described [16]. Briefly, cells were washed three times with the non-supplemented medium and then further incubated for 45 min with PrestoBlue™ reagent (Life Technologies, Carlsbad, CA, USA) diluted in the supplemented medium. Fluorescence was measured (excitation 560 nm; emission 590 nm) on a Synergy HT Multi-Mode Microplate Reader (BioTek Instruments Inc., Winooski, VT, USA) in five technical replicates, excluding the background. For cell cycle profile analysis, cells were plated on 6-well plates (10^5^ cells/well) and incubated for 48 h. Following trypsinization, cell pellets were fixed in 70% ice-cold ethanol for at least 12 h and subsequently incubated with 0.1 mg/mL RNase A (Ambion, Austin, TX, USA) and 5 μg/mL propidium iodide (Sigma, Livonia, MI, USA) in PBS for 30 min in the dark. Cellular DNA content for cell cycle distribution analysis was carried out with flow cytometry using a BD Accuri cytometer (BD Biosciences, Belgium, Germany), plotting at least 20,000 events per sample. The percentage of cells in the different phases of the cell cycle was determined using FlowJo 7.6.5 software (Tree Star, Inc., Orlando, FL, USA), after the exclusion of cell debris and aggregates [81].

### 4.9. Immunofluorescence Analysis

U87 cells (grown for 48 h on glass slides or following cytospin at 400 rpm for 3 min) were fixed for 30 min in 4% paraformaldehyde in PBS. For actin staining, cells were permeabilized and blocked with 0.1% saponin (Sigma) and 0.1% BSA (MD Millipore-Merck, Burlington, MA, USA) in PBS–Tween (PBS-T) and incubated for 1 h with Flash Phalloidin Green 488 (1:100; BioLegend, San Diego, CA, USA) in the dark [16]. For LRP1B expression analysis, cells were permeabilized with 0.5% Triton X-100 in PBS, blocked with 3% BSA and 0.1% Triton X in PBS, and incubated with LRP1B antibody (1:500 in blocking buffer; Sigma SAB4200326) overnight at 4 °C. After washing with PBS, slides were further incubated with secondary anti-rabbit antibody (Alexa 594, Invitrogen A-11037) for 1h at room temperature. All slides were incubated with DAPI (1:1000) for 5 min, washed, and mounted with Vectashield antifade mounting medium (Vector Laboratories). Fluorescence was analyzed using LEICA DM 2000 or with Zeiss Axio Imager Z1 Apotome microscopes with a coupled camera. Nuclear morphometric analysis (NMA) was carried out in images from DAPI-stained nuclei (with at least 300 dpi) using ImageJ analysis software with NII Plugin [82].

### 4.10. CAM Assay

The chicken embryo CAM model was used to evaluate tumor growth and angiogenesis in vivo [83,84,85]. According to the European Directive 2010/63/EU, no ethical approval is needed, and the Portuguese law on animal welfare does not restrict the use of chicken eggs for experimental purposes. Briefly, fertilized chick eggs (*Gallus gallus*) were grown horizontally at 37.5 °C in a humidified atmosphere. On embryonic development day 3 (EDD3), eggs were prepared (window opening) to allow detachment of the developing CAM from the shell, resealed with a transparent adhesive tape, and returned to the incubator. On EDD9, 1 × 10^6^ cells of each condition (resuspended in 5 µL Matrigel) were inoculated per embryo into a 5 mm silicone ring under sterile conditions on top of the growing CAMs. Eggs were resealed and returned to the incubator until EDD13. At the endpoint, embryos were fixed with 4% formaldehyde for 1 h, and CAMs were excised and photographed “ex ovo” under a stereoscope at 20× magnification (Olympus, Tokyo, Japan, SZX16 coupled with a DP71 camera). The tumor size (area) was determined using the “Cell A Olympus” program, and the number of vessels (< 20 µm) growing towards the inoculation site (delimited by the ring) was quantified, as previously described [83,84,85].

### 4.11. Collection of Secretome

U87 control and B9, E6, and H7 cells were grown in 6-well plates (100,000 cells/well) in supplemented medium. After 48 h in culture, cells were serum-deprived and maintained for another 48 h, after which the conditioned medium (secretome) was collected, centrifuged, and filtered. Protein quantification of each secretome was performed with the DC protein assay kit (Bio-Rad Laboratories, Hercules, CA, USA), according to the manufacturer’s protocol, and 100 µg of protein was used for proteome analysis using LC-MS.

### 4.12. Proteomic Evaluation Using Liquid Chromatography–Mass Spectrometry

Protein from each sample (100 µg) was processed for proteomic analysis following a solid-phase-enhanced sample preparation (SP3) protocol, as described in [86]. Enzymatic digestion was performed with 2µg trypsin/LysC overnight at 37 °C at 1000 rpm. The resulting peptide concentration was measured with fluorescence. Protein identification and quantitation were performed using nanoLC-MS/MS equipment [87] composed of an Ultimate 3000 liquid chromatography system coupled to a Q-Exactive Hybrid Quadrupole-Orbitrap mass spectrometer (Thermo Scientific, Bremen, Germany), as previously described [87]. The mass spectrometry proteomics data were deposited to the ProteomeXchange Consortium via the Proteomics Identification Database (PRIDE) partner repository [88], as indicated in the *Data Availability Statement.*

### 4.13. Statistical Analysis

In vitro results are presented as mean ± SEM of at least 3 independent experiments (unless otherwise stated). Student *t*-test was performed to evaluate the statistical significance between different clones versus control. In vivo CAM assay results were statistically analyzed with the ANOVA test (followed by Dunnett’s test). All Values of *p* < 0.05 were considered statistically significant.

## 5. Conclusions

In conclusion, using a dual sgRNA CRISPR/Cas9 strategy, we were able to effectively edit *LRP1B* in U87 cells, generating different LRP1B-altered cell clones. This approach allowed us to investigate the impact of LRP1B loss on GB cell biology and growth, both in vitro and in vivo. Additionally, our research uncovered alterations in the cellular secretome and differential expression of proteins which were not previously linked to LRP1B.

The implications of our study may extend to applied research. On the one hand, the novel cellular tools developed in this study hold potential for advancing our understanding of the role of LRP1B in cancer. These tools open up new avenues for future research and provide opportunities for further discoveries, with potential for extension to other cancer models beyond GB. On the other hand, the disclosure of potential new players associated with LRP1B presents exciting prospects for exploring them as therapeutic targets and strategies for addressing glioblastoma and other types of cancer. While directly modifying the *LRP1B* gene in cancer cells may present challenges, targeting the molecules influenced by LRP1B could offer more suitable avenues for therapeutic intervention.

Overall, our study not only sheds light on the functional consequences of LRP1B alterations in GB but also paves the way for future investigations into its broader implications in cancer research and the development of innovative therapeutic approaches.

## Figures and Tables

**Table 1:** Summary of the alterations found in the three selected clones.

Target Exon	Clone	Deletion Length (bp)	Predicted Effect on *LRP1B* mRNA
1	B9	94 (precise deletion)	Loss of the putative Kozac consensus sequence ^a^ and the canonical AUG start codon
		93 (1 bp insertion)	Loss of the putative Kozac consensus sequence ^a^ and the canonical AUG start codon
	E6	26 (1 bp deletion in sgRNA1-binding region) and 25 bp deletion in the sgRNA2-binding region)	Loss of the short consensus sequence at exon 1–intron 1 junction (5′ splice site)
	H7	92 (2 bp insertion)	Loss of the putative Kozac consensus sequence ^a^ and the canonical AUG start codon
		84 (20 bp insertion)	Preservation of the putative Kozac consensus sequence ^a^ and the canonical AUG start codon
85	B9	No deletion	*NA*
	E6	2 bp deletion in the sgRNA4-binding region	Introduction of an ORF-interrupting PTC
	H7	1 bp deletion in the sgRNA4-binding region2 bp deletion in the sgRNA4-binding region	Introduction of an ORF-interrupting PTC

^a^ The first AUG start codon occurs in the context of the so-called Kozak consensus sequence, which functions as the protein translation initiation site (TIS) in most eukaryotic mRNA transcripts [41]. TIS Miner [42] and NetStart 1.0 [43] softwares were used to predict the putative TIS. Nevertheless, is important to note that alterations in exon 1 could potentially impact the overall effect on *LRP1B* mRNA. *NA*—non-applicable.

## Data Availability

The mass spectrometry proteomics data were deposited to the ProteomeXchange Consortium via the Proteomics Identification Database (PRIDE) partner repository [88] with the dataset identifier PXD042667.

## Supplementary Materials

The following supporting information can be downloaded at https://www.mdpi.com/article/10.3390/ijms241411285/s1.
