# Supplementary material for: Using a Dual CRISPR/Cas9 Approach to Gain Insight into the Role of LRP1B in Glioblastoma
Source: Int J Mol Sci. 2023 Jul 10;24(14):11285. doi: 10.3390/ijms241411285 (PMC10379115; doi:10.3390/ijms241411285)
Supplement: Supplementary file 1 [file ijms-24-11285-s001.zip › ijms-2460790-supplementary.pdf]

International Journal of *Molecular Sciences*

MDPI

*Supplementary Materials*

# Using a Dual CRISPR/Cas9 Approach to Gain Insight into the Role of LRP1B in Glioblastoma

**Joana Peixoto [1,2 #], Catarina Príncipe [1,2,3 #], Ana Pestana [1,2, †], Hugo Osório [1,4,5], Marta Teixeira Pinto [1,4], Hugo Prazeres [1,4], Paula Soares [1,2,4,5], Raquel T. Lima [1,2,4,5*]**

## Supplementary Tables

**Table S1. sgRNA target sequences used and their respective *off-* and *on-target* scores**

| Target exon | Position within the gene (bp)[a] | Strand | sgRNA ID | sgRNA target sequence (5′ → 3′) | PAM sequence | Off-target score[b] | On-target score[c] |
|---|---|---|---|---|---|---|---|
| 1 | 6720 | Minus | sgRNA1 | GACATTGTGGTCGCCCGGTA | AGG | 96.6 | 51.9 |
| | 6800 | Plus | sgRNA2 | CGTGGGAGCCGACCGAGGTA | AGG | 90.2 | 50.3 |
| 85 | 1 862 943 | Minus | sgRNA3 | TTTGGTCCTTCATAGCGCGT | TGG | 94.8 | 32.1 |
| | 1 862 993 | Minus | sgRNA4 | TTATAATGCAGTGCCCCCCA | TGG | 80.5 | 44.2 |

[a] Human LRP1B RefSeqGene NG_051023.1, NCBI Reference Sequence [32]. [b] Hsu et al. [37]. [c] Doench et al. [57]. Off- and on-target scores range from 0 to 100, with higher scores indicating lower off-target potential and higher predicted Cas9 activity at the target site, respectively.

**Table S2. Potential off-target sequences within the human genome for each selected sgRNA**

| sgRNA Name | Sequence | PAM | Score | Gene | Locus |
|---|---|---|---|---|---|
| sgRNA 1 | GACATTGTGGTCGCCCGGTA | AGG | 100.0 | LRP1B (ENSG00000168702) | Chr2:+142888311 |
| | TAGAGTGTGGTCGCCCCGTA | CGG | 0.7 | | Chr9:-98949462 |
| | GAAATCGTGCTCGCCCAGTA | GGG | 0.4 | METTL23 (ENSG00000181038) | Chr17:-74723151 |
| | GACATTGTGGTCACCAGGTA | AGG | 0.4 | | Chr11: +121529897 |
| | GACAGGGTGGGCGCCCGGTG | CGG | 0.3 | UGCG (ENSG00000148154) | Chr9: -114659411 |
| | GACATGGAAGTCTCCCGGTA | TAG | 0.2 | | Chr2: -14296956 |
| | GCCATTGTGGAAGCCCTGTA | AGG | 0.2 | | Chr11: +121358605 |
| | GACATTGTGATCGCCAGGAA | GGG | 0.1 | | Chr5: -5572203 |
| | GTCATTGTGTTAGCCTGGTA | AGG | 0.1 | | Chr13:+44381193 |
| | GACAATGAGGTCGCCAGGCA | AGG | 0.1 | | Chr22: +28198148 |
| | GAGATGGTGGCCGCCTGGTA | GGG | 0.1 | | Chr21:+46184543 |
| sgRNA 2 | CGTGGGAGCCGACCGAGGTA | AGG | 100.0 | LRP1B (ENSG00000168702) | Chr2:+142888217 |
| | GGAGGGAGCGCACCGAGGTA | CAG | 0.9 | | Chr16:+2683345 |
| | GGAGGGAGCGCACCGAGGTA | CAG | 0.9 | | Chr16:+2616850 |
| | CGAAAGAGCCGGCCGAGGTA | GAG | 0.8 | | Chr15:+82042456 |
| | CCTGGGAACAGACCGAGGGA | GAG | 0.6 | | Chr19:+4454107 |
| | CGAAGGGGCCGACCGAGGTC | AAG | 0.6 | | Chr9:+34376639 |
| | CTTGGCAGCTGCCCGAGGTA | GAG | 0.5 | | Chr2:+67487160 |
| | CGGAGGAGCCGGCCGAGGTG | CAG | 0.4 | | Chr2:+219922477 |
| | CGTCCGAGCCGCCCGAGGTG | GAG | 0.4 | FOXO4 (ENSG00000184481) | ChrX:+70316526 |
| | TGTGGGAGCTGCCCGAGGTT | GAG | 0.4 | GNG7 (ENSG00000176533) | Chr19:+2513099 |
| | CGTGGGAGCCGACCGAGGTA | TAG | 0.4 | | Chr7:+157252678 |
| sgRNA 3 | TTTGGTCCTTCATAGCGCGT | TGG | 100.0 | LRP1B (ENSG00000168702) | Chr2:+141032088 |
| | GATGGTCTTTTATAGCGCGT | AAG | 0.9 | | Chr15:+60777206 |
| | GTTTGTCATTCATAGCCCGT | GAG | 0.8 | | Chr10:+77379183 |
| | TTGGGCCTTTCATAGCACGT | AGG | 0.4 | | Chr18:+47828723 |
| | TTTGCTCTTTCATAGCACCT | TAG | 0.2 | | Chr1:-144870905 |
| | TTTGTTCTTTCATAGCACAT | TGG | 0.2 | | Chr3:+29951857 |
| | TCTGGTCCTCCATAGCTCTT | GGG | 0.2 | | Chr8: +22995922 |
| | TCTGGTCCTGCATAGCTCTT | GGG | 0.2 | | Chr8:-22938350 |
| | TGTGTTCCTTCATAGCGGGA | TGG | 0.2 | | Chr10:+32865797 |
| | TTTGGTCAATCACAGCGCGC | CAG | 0.2 | | Chr9:+33076306 |
| | TTTGGCCATTCTTAGCGCCT | GAG | 0.2 | | Chr14: -33848061 |
| sgRNA 4 | TTATAATGCAGTGCCCCCCA | TGG | 100.0 | LRP1B (ENSG00000168702) | Chr2:+141032038 |
| | TTCCCATCCAGTGCCCCCCA | CAG | 1.4 | | Chr20:+59999886 |
| | TTGCCATGCTGTGCCCCCCA | AAG | 1.3 | | Chr10:-130874277 |
| | CTATAATACAGTTCCCCCCA | AAG | 1.3 | | Chr7:-13572699 |
| | TTAGAATGCAGTGACCCCCA | AGG | 1.3 | | ChrY:-16857284 |
| | TCACAAAGCCGTGCCCCCCA | GAG | 1.0 | | Chr20:+29596954 |
| | TTCTAATCCAGTGCTCCCCA | AGG | 0.9 | | Chr5:+107228315 |
| | GGCTACTGCAGTGCCCCCCA | AGG | 0.8 | | Chr13:+113761336 |
| | TTCTTCTCCAGTGCCCCCCA | TGG | 0.8 | MICB (ENSG00000204516) | Chr6: +31474805 |
| | TTCTTCTCCAGTGCCCCCCA | TGG | 0.8 | PERB11.1 (ENSG00000204520) | Chr6:+31379730 |
| | TGAGAAAGCAGTGCCCTCCA | AGG | 0.5 | | Chr2:+109576512 |

Only the the first 10 off-target sequences for each sgRNA are presentes, ordered based on their Score values. In these sequences, mismatches are denoted by red letters.

**Table S3. Oligonucleotides used for sgRNA cloning**

| Oligonucleotide duplex name | Oligonucleotide name | Sequence (5′ → 3′) |
|---|---|---|
| Duplex-1 | 1-sense | **CACC**GACATTGTGGTCGCCCGGTA |
| | 1-antisense | **AAAC**TACCGGGCGACCACAATGTC |
| Duplex-2 | 2-sense | **CACC**GCGTGGGAGCCGACCGAGGTA |
| | 2-antisense | **AAAC**TACCTCGGTCGGCTCCCACGC |
| Duplex-3 | 3-sense | **CACC**GTTTGGTCCTTCATAGCGCGT |
| | 3-antisense | **AAAC**ACGCGCTATGAAGGACCAAAC |
| Duplex-4 | 4-sense | **CACC**GTTATAATGCAGTGCCCCCCA |
| | 4-antisense | **AAAC**TGGGGGGCACTGCATTATAAC |

**Table S4. Oligonucleotide primers used for genomic DNA analysis**

| Oligonucleotide name | Position within the gene (bp)[a] | Strand | Sequence (5′ → 3′) |
|---|---|---|---|
| LRP1B-01-Fw | 6574 | Plus | GCTGACTGGCTGGACTCATT |
| LRP1B-01-Rv | 6869 | Minus | TTATCTGCAAGCATCGCCCA |
| LRP1B-01-In | 6774 | Minus | ACCCTGGCAATCGGCAATAA |
| LRP1B-85-Fw | 1 862 483 | Plus | AGGTGTGAAGGAGGCAACAA |
| LRP1B-85-Rv | 1 862 145 | Minus | GCAATGGGCACAATACGGAA |
| LRP1B-85-In | 1 862 966 | Minus | ACCTTACACACTTGTCAACCTCA |

[a] Human *LRP1B* RefSeqGene NG_051023.1, NCBI Reference Sequence [32].

**Table S5. List of differentially expressed proteins (DESPs) found in the secretomes of clones B9, E6 and H7, when compared to secretomes from mock cells.**

| | | Accession | Description | Gene Symbol | Abundance Ratio: (clone) / Mock | Abundance Ratio Adj. P-Value: (clone) / Mock |
|---|---|---|---|---|---|---|
| Clone B9 *vs* Mock | Up-regulated | Q9UNZ2 | **NSFL1 cofactor p47** | NSFL1C | 100 | 1.7206E-16 |
| | | Q96KG7 | **Multiple epidermal growth factor-like domains protein 10** | MEGF10 | 100 | 1.7206E-16 |
| | | P05231 | **Interleukin-6** | IL6 | 100 | 1.7206E-16 |
| | | P08254 | **Stromelysin-1** | MMP3 | 11.524 | 2.6517E-05 |
| | | P48307 | **Tissue factor pathway inhibitor 2** | TFPI2 | 9.668 | 0.0002 |
| | | P03956 | **Interstitial collagenase** | MMP1 | 9.409 | 0.0002 |
| | | Q7Z304 | **MAM domain-containing protein 2** | MAMDC2 | 8.76 | 0.0004 |
| | | P21741 | **Midkine** | MDK | 7.771 | 0.0013 |
| | | P17301 | **Integrin alpha-2** | ITGA2 | 6.729 | 0.0045 |
| | | P05120 | **Plasminogen activator inhibitor 2** | SERPINB2 | 6.53 | 0.0057 |
| | | Q16363 | **Laminin subunit alpha-4** | LAMA4 | 6.387 | 0.0068 |
| | Down - regulated | P02452 | **Collagen alpha-1(I) chain** | COL1A1 | 0.361 | 0.0379 |
| | | P58215 | **Lysyl oxidase homolog 3** | LOXL3 | 0.359 | 0.0366 |
| | | P41222 | **Prostaglandin-H2 D-isomerase** | PTGDS | 0.317 | 0.0152 |
| | | Q13449 | **Limbic system-associated membrane protein** | LSAMP | 0.308 | 0.0120 |
| | | P21810 | **Biglycan** | BGN | 0.288 | 0.0075 |
| | | P08123 | **Collagen alpha-2(I) chain** | COL1A2 | 0.286 | 0.0071 |
| | | P29279 | **CCN family member 2** | CCN2 | 0.269 | 0.0044 |
| | | P05156 | **Complement factor I** | CFI | 0.258 | 0.0032 |
| | | Q9BRK3 | **Matrix remodeling-associated protein 8** | MXRA8 | 0.254 | 0.0028 |
| | | P05109 | **Protein S100-A8** | S100A8 | 0.228 | 0.0011 |
| | | Q9BQT9 | **Calsyntenin-3** | CLSTN3 | 0.221 | 0.00081 |
| | | Q9UI42 | **Carboxypeptidase A4** | CPA4 | 0.21 | 0.00051 |
| | | P06276 | **Cholinesterase** | BCHE | 0.185 | 0.0001 |
| | | Q9BXX0 | **EMILIN-2** | EMILIN2 | 0.156 | 2.4687E-05 |
| | | P01009 | **Alpha-1-antitrypsin** | SERPINA1 | 0.149 | 1.5161E-05 |
| | | Q14126 | **Desmoglein-2** | DSG2 | 0.133 | 4.1205E-06 |
| | | Q8IX30 | **Signal peptide. CUB and EGF-like domain-containing protein 3** | SCUBE3 | 0.01 | 1.7206E-16 |
| Clone E6 *vs* Mock | Up-regulated | Q96KG7 | **Multiple epidermal growth factor-like domains protein 10** | MEGF10 | 100 | 1.7171E-16 |
| | | P05231 | **Interleukin-6** | IL6 | 100 | 1.7171E-16 |
| | | Q9C0C2 | **182 kDa tankyrase-1-binding protein** | TNKS1BP1 | 100 | 1.71716E-16 |
| | | Q9UNZ2 | **NSFL1 cofactor p47** | NSFL1C | 100 | 1.71716E-16 |

**Table S5.** *(cont.)*

| | | Accession | Description | Gene Symbol | Abundance Ratio: (clone) / Mock | Abundance Ratio Adj. P-Value: (clone) / Mock |
|---|---|---|---|---|---|---|
| **Clone E6 *vs* Mock** | Down - regu- lated | P21589 | **5'-nucleotidase** | NT5E | 0.173 | 0.0400 |
| | | Q13449 | **Limbic system-associated membrane protein** | LSAMP | 0.139 | 0.013337294 |
| | | Q9BXX0 | **EMILIN-2** | EMILIN2 | 0.1 | 0.002047735 |
| **Clone H7 *vs* Mock** | Up- regu- lated | P05231 | **Interleukin-6** | IL6 | 100 | 1.5956E-16 |
| | | Q9UNZ2 | **NSFL1 cofactor p47** | NSFL1C | 100 | 1.5956E-16 |
| | | Q96KG7 | **Multiple epidermal growth factor-like domains protein 10** | MEGF10 | 100 | 1.5956E-16 |
| | | Q9C0C2 | **182 kDa tankyrase-1-binding protein** | TNKS1BP1 | 100 | 1.5956E-16 |
| | | P36222 | **Chitinase-3-like protein 1** | CHI3L1 | 17.201 | 1.25923E-07 |
| | | Q15782 | **Chitinase-3-like protein 2** | CHI3L2 | 10.62 | 3.37175E-05 |
| | | Q7Z304 | **MAM domain-containing protein 2** | MAMDC2 | 9.764 | 7.90558E-05 |
| | | P00751 | **Complement factor B** | CFB | 9.3 | 0.0001 |
| | | P43235 | **Cathepsin K** | CTSK | 7.39 | 0.0011 |
| | | P06702 | **Protein S100-A9** | S100A9 | 7.346 | 0.0011 |
| | | P05156 | **Complement factor I** | CFI | 7.108 | 0.0015 |
| | | Q9BU40 | **Chordin-like protein 1** | CHRDL1 | 6.741 | 0.0024 |
| | | P49908 | **Selenoprotein P** | SELENOP | 6.686 | 0.0025 |
| | | P05109 | **Protein S100-A8** | S100A8 | 6.415 | 0.0036 |
| | | Q9UKU9 | **Angiopoietin-related protein 2** | ANGPTL2 | 5.138 | 0.0186 |
| | | Q15274 | **Nicotinate-nucleotide pyrophosphorylase [carboxylating]** | QPRT | 4.797 | 0.0297 |
| | | Q7Z7M9 | **Polypeptide N-acetylgalactosaminyltransferase 5** | GALNT5 | 4.771 | 0.0307 |
| | | Q16363 | **Laminin subunit alpha-4** | LAMA4 | 4.705 | 0.0338 |
| | Down- regu- lated | P55290 | **Cadherin-13** | CDH13 | 0.305 | 0.0365 |
| | | O95388 | **CCN family member 4** | CCN4 | 0.294 | 0.0287 |
| | | P29279 | **CCN family member 2** | CCN2 | 0.273 | 0.0173 |
| | | Q99715 | **Collagen alpha-1(XII) chain** | COL12A1 | 0.253 | 0.0101 |
| | | O15240 | **Neurosecretory protein VGF** | VGF | 0.194 | 0.0012 |
| | | Q96AM1 | **Mas-related G-protein coupled receptor member F** | MRGPRF | 0.19 | 0.0010 |
| | | P15692 | **Vascular endothelial growth factor A** | VEGFA | 0.167 | 0.0003 |
| | | Q13449 | **Limbic system-associated membrane protein** | LSAMP | 0.144 | 7.52018E-05 |
| | | Q9BXX0 | **EMILIN-2** | EMILIN2 | 0.113 | 5.36226E-06 |
| | | P21810 | **Biglycan** | BGN | 0.1 | 1.29275E-06 |
| | | Q8N6T3 | **ADP-ribosylation factor GTPase-activating protein 1** | ARFGAP1 | 0.01 | 1.5956E-16 |
| | | Q9NT68 | **Teneurin-2** | TENM2 | 0.01 | 1.5956E-16 |
| | | O75635 | **Serpin B7** | SERPINB7 | 0.01 | 1.5956E-16 |
| | | Q86UN3 | **Reticulon-4 receptor-like 2** | RTN4RL2 | 0.01 | 1.5956E-16 |

## Supplementary Figures

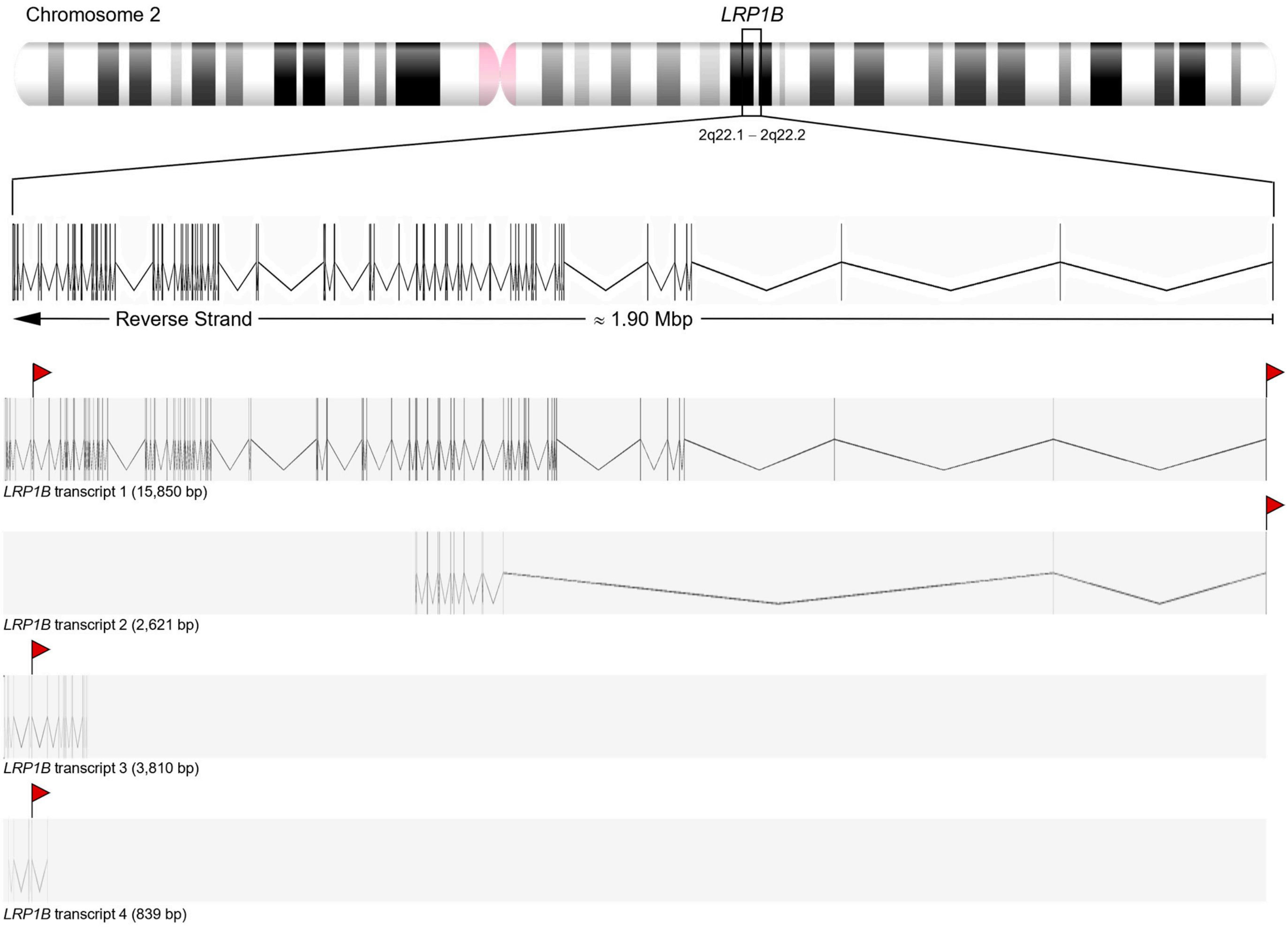


**Figure S1. Schematic representation of *LRP1B* gene and targeted regions.** Chromosomal location of the *LRP1B* locus. The chromosome ideogram was obtained from the NCBI online tool [31]. Human *LRP1B* is located on the long arm of chromosome 2 at position 2q22.1-2q22.2. It spans from 140.231.423 to 142.131.016 bp, according to the human genome assembly GRCh38.p13 from the Genome Reference Consortium [78] being approximately 1.90 megabase pairs (Mbp) long and composed of 91 exons that comprise 13.80 kilobase pairs (kbp) of coding sequence (CDS). Schematic representation of *LRP1B* gene structure depicting the exon-intron structure of *LRP1B*. The human *LRP1B* (ID: ENSG00000168702) has four putative protein-coding transcripts [33]: (i) full-length *LRP1B* transcript (***LRP1B* transcript 1**; ID: ENST00000389484.8), which includes all 91 annotated exons that together encode a 4599-amino acid membrane-bound protein (LRP1B) and (ii) three other predicted alternatively spliced *LRP1B* transcripts (***LRP1B* transcripts 2, 3** and **4**) that have their CDS incomplete on the 3′ end, 5′ end, or both ends. Specifically, *LRP1B* transcript 2 contains 14 exons (1,2,18-29; ID: ENST00000434794.1), which encode a putative 781-amino acid soluble LRP1B isoform. *LRP1B* transcripts 3 and 4 encode two putative membrane-bound LRP1B isoforms of 789 and 280 amino acids, respectively. *LRP1B* transcript 3 contains 17 exons (74-89, 91; ID: ENST0000043-7977.5), whereas *LRP1B* transcript 4 contains 7 exons (84-89. including an extra 114 bp exon between exons 86 and 87; ID: ENST00000442974.1). The gene and transcripts schemes were obtained from Ensembl release version 100 [33,35] The most upstream exons shared by the majority of the *LRP1B* predicted transcripts are signaled with red flags.

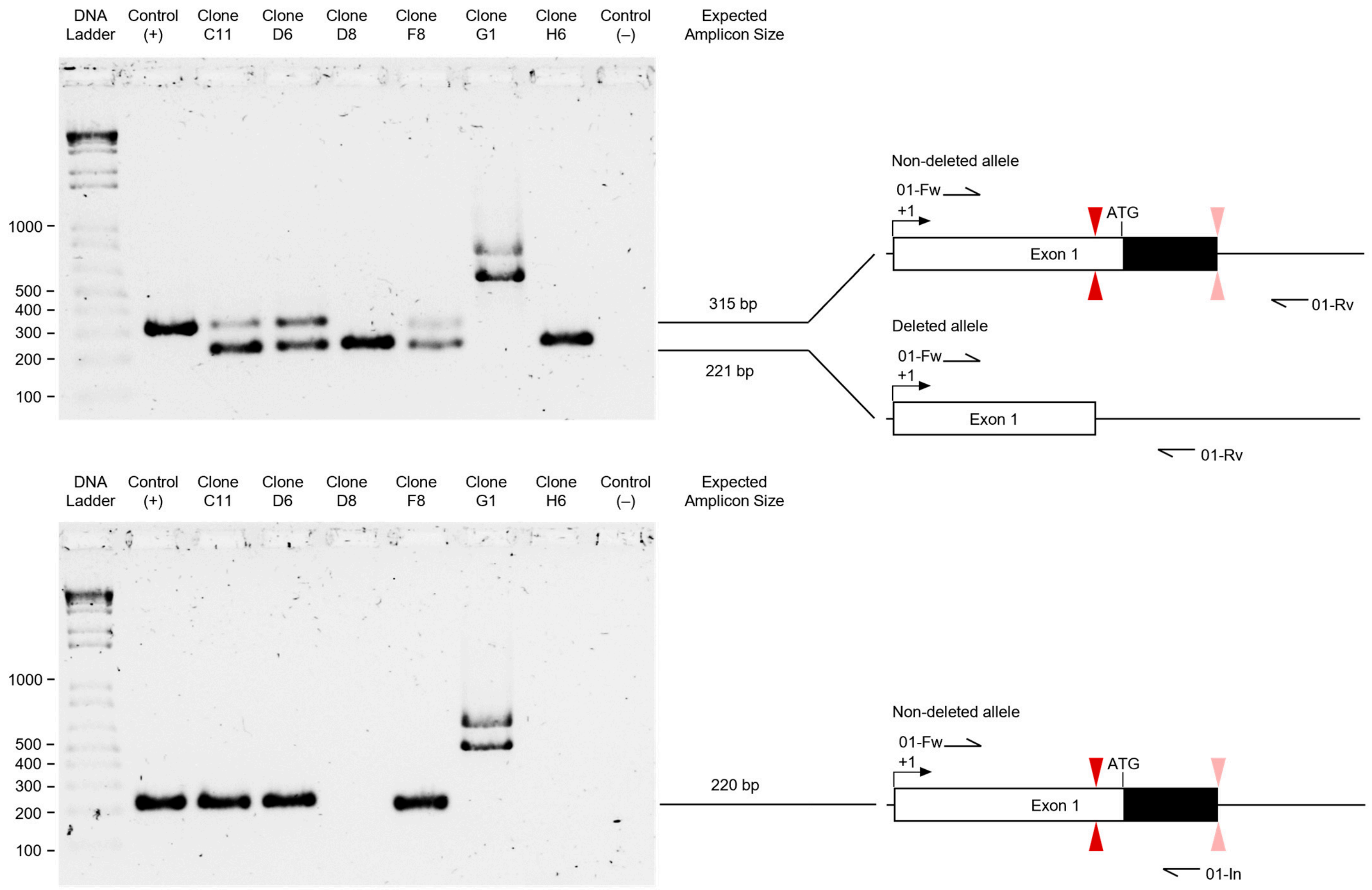


**Figure S2. Representative images of PCR analysis of transfected selected clones**. Exon 1 analysis for some clones derived from U87 cells transfected with paired PX459-sgRNA1 and -2 vectors. *Upper panel*. the CRISPR/Cas9-targeted exon 1 was amplified from genomic DNA using the primers forward (01-Fw) and reverse (01-Rv). Expected sizes of the PCR amplicons for *LRP1B* non-deleted and deleted alleles are 315 bp and 221 bp, respectively. *Lower panel*. the CRISPR/Cas9-targeted exon 1 was amplified from genomic DNA using the primers forward (01-Fw) and reverse internal (01-In). The expected size of the PCR amplicon for the *LRP1B* non-deleted allele is 220 bp. Control (+), positive control using genomic DNA from the parental (wild-type) U87 cells and the appropriate primer set for each PCR; Control (-), negative control for each primer set (no DNA template); DNA Ladder, 1 kbp DNA ladder. Schematic representation of the *LRP1B* non-deleted and deleted alleles is depicted on the right side of the panels. Labeled black arrows indicate the positions and orientations of PCR primers. The arrowheads indicate the CRISPR/Cas9 target sites within *LRP1B* exon 1.

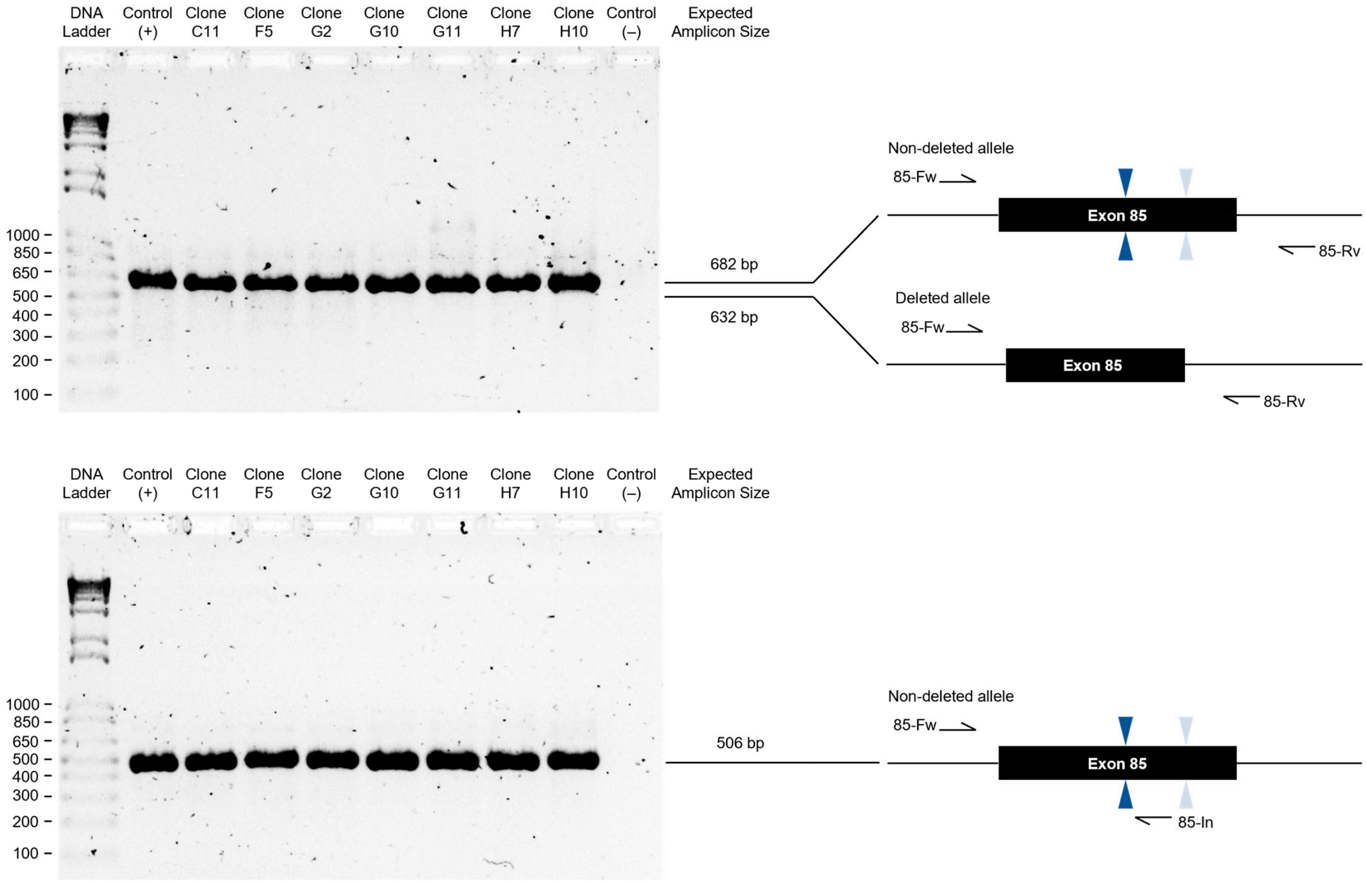


**Figure S3. Representative images of PCR analysis of exon 85 for some clones derived from U87 cells transfected clones** with A) PX459-sgRNA3 and PX459-sgRNA4 vectors and B) all PX459-sgRNA vectors (1. -2. -3 and -4 ). Upper panels. the CRISPR/Cas9-targeted exon 85 was amplified from genomic DNA using the primers forward (85-Fw) and reverse (85-Rv). The expected sizes of the PCR amplicons for *LRP1B* non-deleted and deleted alleles are 682 bp and 632 bp, respectively. Lower panel. the CRISPR/Cas9-targeted exon 85 was amplified from genomic DNA using the primers forward (85-Fw) and reverse internal (01-In). The expected size of the PCR amplicon for the *LRP1B* non-deleted allele is 506 bp. Control (+), positive control using genomic DNA from the parental U87 human glioblastoma cell line and the appropriate primer set for each PCR; Control (-), negative control for each primer set (no DNA template); DNA Ladder, 1 kbp DNA ladder. Schematic representation of the *LRP1B* non-deleted and deleted alleles is depicted on the right side of the panels. Labeled black arrows indicate the positions and orientations of PCR primers. The arrowheads indicate the CRISPR/Cas9 target sites within *LRP1B* exon 85.

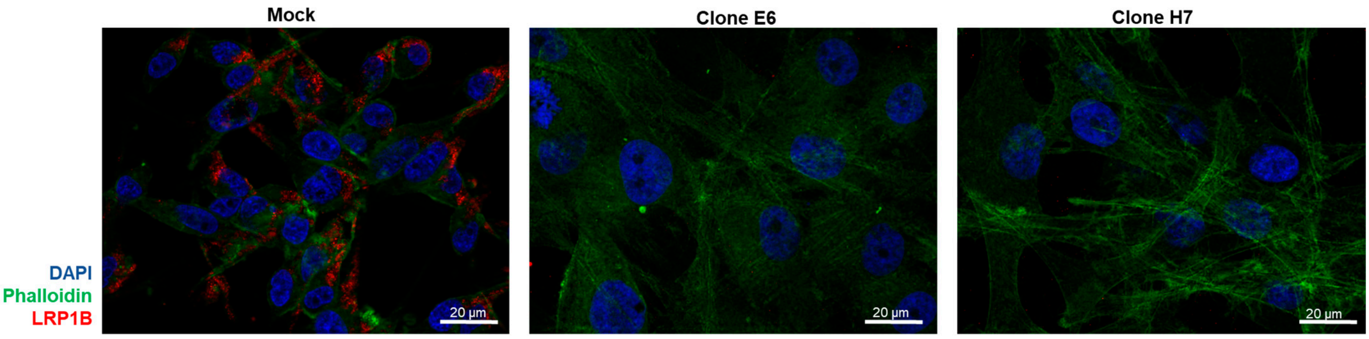


**Figure S4. Decreased LRP1B expression in LRP1B-edited clones E6 and H7, compared to mock cells, evaluated by immunofluorescence.** Images are representative of 2 independent experiments.

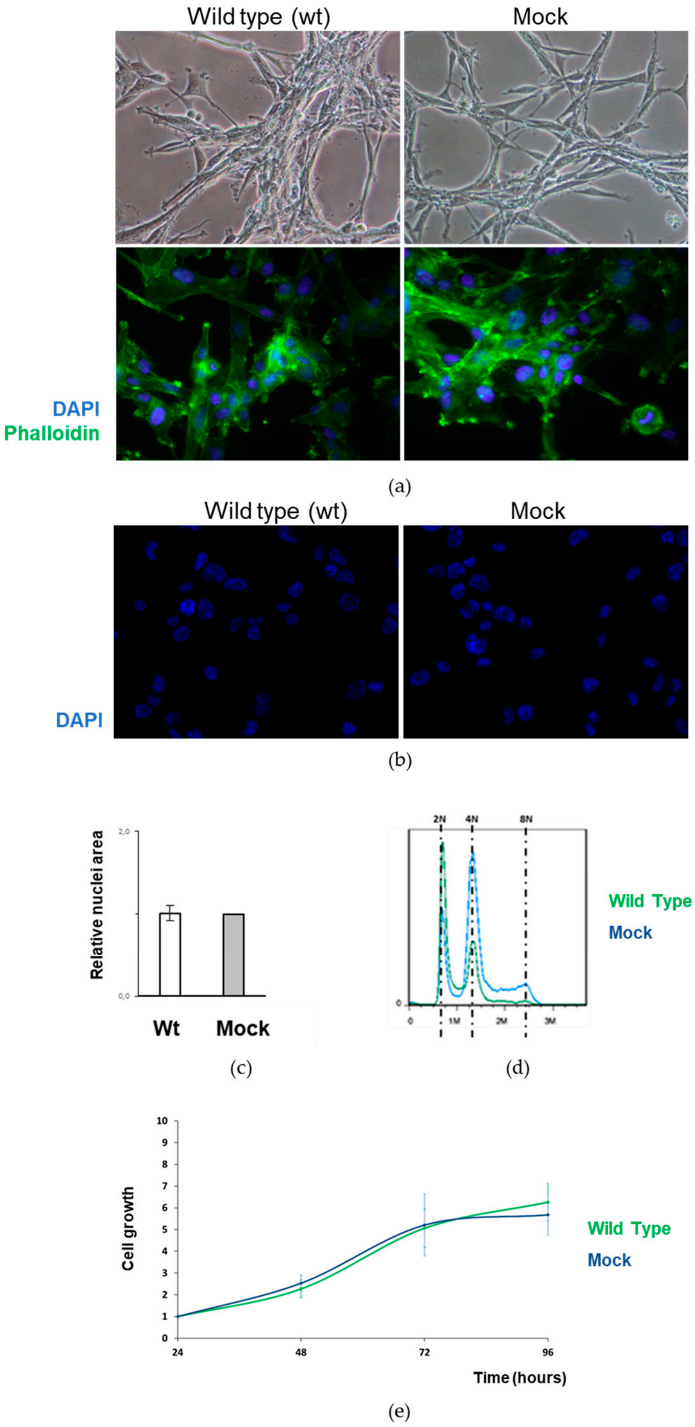


**Figure S5. U87 wild type (wt; non-transfected) and mock cells.** (a) Cell morphology in adherent cells in culture evaluated by phase contrast microscopy and fluorescence microscopy (b) Representative images of DAPI-stained nuclei from cells after cytospin. Cell nuclei area (evaluated with ImageJ software with NII plugin) was analyzed in relation to mock cells nuclei area. Results are the mean ± SEM of 3 independent experiments. (d**)** Representative cell cycle profile evaluated by flow cytometry following propidium iodide staining. Images are representative of, at least, 3 independent experiments. (e) Cell growth analysis, with Presto blue viability assay following 24 h, 48, 72, and 96 h in culture. Results, expressed in relation to the 24 h, are the mean ± SEM of at six independent experiments.
